# Adaptive Steered Molecular Dynamics Combined With Protein Structure Networks Revealing the Mechanism of Y68I/G109P Mutations That Enhance the Catalytic Activity of D-psicose 3-Epimerase From *Clostridium Bolteae*

**DOI:** 10.3389/fchem.2018.00437

**Published:** 2018-09-24

**Authors:** Jingxuan Zhu, Yi Li, Jinzhi Wang, Zhengfei Yu, Ye Liu, Yi Tong, Weiwei Han

**Affiliations:** ^1^Key Laboratory for Molecular Enzymology and Engineering of Ministry of Education, School of Life Science, Jilin University, Changchun, China; ^2^COFCO (Jilin) Bio-Chemical Technology Co., Ltd, Changchun, China

**Keywords:** D-fructose, D-psicose 3-epimerase (DPEase), catalytic efficiency, molecular modeling, adaptive steered molecular dynamics, protein structure networks

## Abstract

The scarcity, richness, and other important physiological functions of D-psicose make it crucial to increase the yield of D-psicose. The production of D-psicose can be accomplished by D-psicose 3-epimerase (DPEase) from *Clostridium bolteae* (*Cb*DPEase) catalyzing the substrate D-fructose. Although the catalytic efficiency of the *Cb*DPEase has been raised via using the site-directed mutagenesis (Y68I/G109P) technique, structure-activity relationship in the wild-type *Cb*DPEase and Y68I/G109P mutant is currently poorly understood. In our study, a battery of molecular modeling methods [homology modeling, adaptive steered molecular dynamics (ASMD) simulations, and Molecular Mechanics/Generalized Born Surface Area (MM-GB/SA)], combined with protein structure networks, were employed to theoretically characterize the reasons for the differences in the abilities of the D-fructose catalyzed by the wild-type *Cb*DPEase and Y68I/G109P mutant. Protein structure networks demonstrated that site-directed mutagenesis enhanced the connectivity between D-fructose and *Cb*DPEase, leading to the increased catalytic efficiency mediated by the functional residues with high betweenness. During the dissociation of the D-fructose from the Y68I/G109P mutant, planes of benzene rings of F248 and W114 could be continuously parallel to the stretching direction of D-fructose. It made the tunnel have an open state and resulted in the stable donor-π interactions between D-fructose and the benzene rings around 18Å. The stronger substrate-protein interactions were detected in the Y68I/G109P mutant, instead of in the wild-type *Cb*DPEase, which were consistent with the binding free energy and Potential Mean of Force (PMF) results. The theoretical results illustrated the reasons that Y68I/G109P mutations increased the catalytic efficiency of *Cb*DPEase and could be provided the new clue for further DPEase engineering.

## Introduction

D-psicose, as a C-3 epimer of D-fructose, has plentiful significant physiological functions, covering anti-hyperglycemia and anti-obesity effects (Granström et al., 2004; Fukada et al., 2010; Iida et al., 2010; Zhang et al., 2016; Shintani et al., 2017). It has a sucrose sweetness of 70%, and contributes less calories for its poor absorption in the digestive tract (Granström et al., 2004; Fukada et al., 2010). D-psicose is therefore commonly applied as a functional sugar in the treatment of diabetes and obesity (Ochiai et al., 2013). Furthermore, D-psicose has various physiological characteristics beneficial to health. For example, it can efficaciously reduce the accumulation of abdominal fat (Matsuo et al., 2001; Ochiai et al., 2014), remove reactive oxygen species (ROS) (Murata et al., 2003), and inhibit hepatic lipase activities (Matsuo et al., 2015). Besides, it can reduce the postprandial glycemic excursion (Matsuo and Izumori, 2006; Hayashi et al., 2010), improve the metabolism of serum lipids (Afach et al., 2008), and protect the nervous system (Takata et al., 2005). Also, it can strengthen insulin resistance (Hossain et al., 2011, 2012), treat atherosclerotic diseases and so on (Murao et al., 2007).

D-psicose is rare in nature, and its chemical synthesis is difficult to get (Zhang et al., 2016). Therefore, the technology of D-psicose production by the enzymatic reaction of ketose 3-epimerase has been widely concerned in commercial production (Kim et al., 2006; Yoshida et al., 2007; Mu et al., 2011; Chan et al., 2012). In general, D-fructose can serve as the substrate to produce the D-psicose and characterize the enzymic activity of the ketose 3-epimerase. D-tagatose 3-epimerase (DTEase) isolated from *Pseudomonas cichorii* ST-24 (*P. cichorii* DTEase) (Itoh et al., 1994; Izumori et al., 2008) is the original member of the ketose 3-epimerases. DTEase family enzymes are derived from a variety of microorganisms and their relative sequences are quite similar. It indicates that enzymes, with the significantly enhanced efficiency for D-psicose production, may have a common catalytic mechanism (Zhang et al., 2013) which catalyzes the bioconversion from D-fructose to D-psicose by the epimerization reaction at the C-3 position. Particularly, DTEase from *Agrobacterium tumefaciens* (*A. tumefaciens* DPEase), characterized by Kim et al. was defined as D-psicose 3-epimerase (DPEase) for it was mainly designed for D-psicose. Before long, puzzles concerning crystal structures of *P. cichorii* DTEase and *A. tumefaciens* DPEase were successively resolved. DPEase is a tetramer composed of four TIM-barrel shaped monomers, and the active site in each monomer differs from other TIM-barrel enzymes. The metal ion (Mn^2+^or Co^2+^) in the monomer of DPEase is coordinated as an octahedron by two water molecules and four residues (E150, D183, H209, and E244) that are entirely conserved in the DPEase family. In the case of D-fructose binding, the substrate replaces water molecules in the active site to form a conformation of the intermediate cis-enediolate. O1, O2, and O3 of the substrate are interacted with four key residues of the active site.

Recently, Jia et al. reported the characterization study of the wild-type *Clostridium bolteae* DPEase (*Cb*DPEase) (Jia et al., 2014). Also, the catalytic activity of the wild-type *Cb*DPEase was promoted by mutations in the Y68 and G109 sites (Zhang et al., 2016). Compared with the wild-type, the Y68I/G109P mutant would be more appropriate for the industrial D-psicose production. To explore the structure–function relationships between the substrate and the wild/mutant type *Cb*DPEase, D-fructose was used as the substrate to investigate the mechanism of Y68I/G109P mutations that enhanced the catalytic activity of *Cb*DPEase by using adaptive steered molecular dynamics and the protein structure networks method.

It was very known that molecular recognition and specific ligand-receptor interactions are critical steps in numerous physiological processes, such as enzyme catalysis, the organelle assembly, and energy conversion. It requires the incorporation of non-covalent bonds between the protein and the ligand to induce the realization of its functional response (Deuflhard et al., 1999). And steered molecular dynamics (SMD) simulation can reveal the details of molecular interactions during dissociation, thus providing important information for the binding mechanism. In our study, adaptive steered molecular dynamics (ASMD) simulations were performed to quantitatively calculated the binding energy of the protein and the ligand, and qualitatively analyzed the protein-ligand interactions and conformational changes of the protein induced by ligand. The dissociation of the substrate is the reverse process of molecular binding and molecular recognition. Hence, the results of ASMD simulations could intuitively reflect the affinity of substrate binding. Besides, by analyzing the protein structure networks, we could figure out characteristics of the dual mutation-induced changes of the connectivity in the networks of the protein-ligand complex. The results of this study may be conductive to the exploration of efficient point mutations in the future, and the results also provided the theoretical basis for the discovery of the novel mechanism of protein design for the DPEase family.

## Materials and methods

### Modeling and validation of dpease, and structure-based sequence alignments

The *Cb*DPEase protein sequence (Genbank ID: EDP19602.1) was obtained from the NCBI server. A similarity search for *Cb*DPEase was performed by the Moddeller 9.20 (Fiser and Sali, 2003) to find potentially related sequences of known structures as suitable templates. Because of the high sequence similarity between *Cb*DPEase and templates (higher than 53%), crystal structures of D-psicose 3-epimerases from *Clostridium cellulolyticum* (*Cc*DPEase) (PDB ID: 3VNK) (Chan et al., 2012) and *Agrobacterium fabrum* (*Af* DPEase) (PDB ID: 2HK1) (Kim et al., 2006) were chosen as the multiple templates for homology modeling. Homology modeling was performed via using Modeler 9.20 (Fiser and Sali, 2003). It generated five models, among which the best one was selected based upon the least DOPE (Discrete Optimized Protein Energy) score (Shen and Sali, 2006). In this rating of the score, a statistical program named the atomic distance-dependent was applied to discern the similarity between templates and models. The lower is the DOPE score, the more reliable is the model (Shen and Sali, 2006). After predicting the three-dimensional structure of *Cb*DPEase, we finished the validation of the modeled structure via using Structure Analysis and Verification Server (SAVS) to assess the overall stereo chemical quality of the modeled protein (www.mbi.ucla.edu/SAVS). Ramachandran plot was analyzed by the program PROCHECK (Laskowski et al., 1993). We further evaluated the modeled structure of PhMTNA by the use of VERIFY3D and ERRAT, a verification algorithm well-suited for the evaluation of the progress of crystallographic model building and refinement (Bowie et al., 1991).

To compare related structures (templates and models) and perform the interactive communication between sequences and structures, the MatchMaker extension of Chimera (Meng et al., 2006) was used for the structure-based sequence alignments and the structures superimposition. The standard Needleman-Wunsch (Needleman and Wunsch, 1970) algorithms BLOSUM-62 and 30% weighting of the secondary structure term (i.e., 70% weighting of the residue similarity term) were employed as the default settings in global and local sequence alignments production, respectively. Based on the structures and their corresponding sequence alignment suitable for Chimera, the AL2CO histogram (Pei and Grishin, 2001) of conservation style was applied to map sequence conservation onto structures with Chimera. And the secondary structure was presented in Pro-origami web service (Stivala et al., 2011) as a cartoon.

### Integrative visual analysis for residue interaction networks of protein-ligand complexes

The Gaussian 09 software (Robb et al., 2009) with B3LYP 6-31+G* level was devoted to optimizing the structure of D-fructose. The optimized structures were illustrated in Figure S6. Autodock vina (Trott and Olson, 2010) was used to dock the D-fructose into the *Cb*DPEase binding pockets, and the lowest and the most stable model was selected for the further simulations.

The latest study of individual residues and their structure-based functions has demonstrated that residue interaction networks (RINs) obtained from three-dimensional structures are promising to investigate the relationship between structures and functions. In RINs, amino acid residues in the protein are referred to as nodes that are connected by edges over the noncovalent interactions identified by Probe (Word et al., 1999). In our study, the RINs for 3D structures of *Cc*DPEase, *Af* DPEase (templates) and *Cb*DPEase were generated from RINerator software (Doncheva et al., 2011). Using the RINalyzer (Doncheva et al., 2011), the resulting networks and accompanying analysis were visualized in Cytoscape (Shannon et al., 2003) and structureViz apps (Morris et al., 2007). The corresponding structures were displayed by UCSF Chimera (Pettersen et al., 2004; Meng et al., 2006). Specifically speaking, the bridge between biological networks visualized in Cytoscape and corresponding 3D structures was acted by the Cytoscape plugin structureViz.

### Molecular dynamics simulation

The D-fructose bound to *Cb*DPEase was performed on 300 ns time scales' molecular dynamics simulation using the Amber 14 software package (Case et al., 2005) with TIP3P water molecules (Harrach and Drossel, 2014). For the ligand (D-fructose), generalized AMBER force field (GAFF) parameters and RESP partial charges (Wang et al., 2004; Maier et al., 2015) were assigned using the ANTECH AMBER program, which shipped with Amber Tools (Sousa da Silva and Vranken, 2012). The production MD was accomplished at 333 K and 1 atm for 60 ps with the desired temperature and pressure (NPT) coupled by Berendsen methods (Darden et al., 1993). The MD trajectory was saved and written to the output file every 2 ps. The RMSD plot in Figure S7 reflected the stabilization of MD simulation for the modeled structure. After MD simulations, the averaged snapshots (within 60–85 ns and 210–265 ns) were chosen to generate the mutant type structures. Y68 was mutated into I68, and G109 was mutated into P109 using Discovery Studio 4.0 client software. The wild-type *Cb*DPEase from 60 to 85 ns and its Y68I/G109P mutant were equilibrated for 10 ns to generate the first two equilibrium trajectories. Besides, the wild-type *Cb*DPEase from 210 to 265 ns and its Y68I/G109P mutant were equilibrated for 10 ns to generate the latter two equilibrium trajectories. They correspond to steps of minimization, heating, density, slowly releasing restraints, and finally an unconstrained molecular dynamics simulation. All the simulations were performed by the same force field and parameter settings as the MD simulations. The trajectories generated from the equilibration were applied in the next Principle component analyses (PCA), network analysis, MM-GB/SA calculations, and CAVER calculations.

### Principle component analyses and free energy landscape analysis

The AMBER14 plugin CPPTRAJ module (Lu and Luo, 2003) was used to perform Principle Component Analyses (PCA) for understanding the most significant conformational changes in the equilibration stage of the wild-type *Cb*DPEase and the Y68I/G109P mutant. All representative structures based on disparate conformational motions were extracted by using OpenGUI interface of VMD (Humphrey et al., 1996), and visualized through PyMOL (Delano, 2002). The energies of macromolecule conformations such as the first two principal components (PC1, PC2) were characterized in the Free Energy Landscape (FEL). By using converting dot distribution to probability distribution (ddtpd) program, the trajectories to the PC1 and PC2 of motion were mapped (Nicolaï et al., 2013; Iida et al., 2016; Tripathi et al., 2016).

### Protein structure network analysis

The network parameters (clusters, hubs, cliques and communities) were utilized to analyze the residue interaction networks. The clusters, and groups of interacting residues (nodes) (the number of interactions is at least two) in the network were calculated by MCODE algorithm (O'Driscoll et al., 2014) visualized in Cytoscape. The hubs are highly connected nodes in the network with at least 4 associated edges on the node. A *k* = n clique is a group of “n” nodes, in which each one is connected to every other node in the group. A community of *k* = n cliques is a collection of cliques that share n-1 nodes among them. The network parameters for hubs, cliques and communities were computed by using the Clique Percolation Method (Palla et al., 2005) when implemented in the CFinder software (Kang et al., 2014). Edge attribute files were generated from RINerator. The protein structures used in the protein network analysis were the representative structures from 2500 snapshots of equilibration trajectories in the wild-type *Cb*DPEase and the Y68I/G109P mutant.

### Network centrality analysis

Network Centrality analysis, one of key features of RINalyzer, can compute weighted centrality measures. It incorporates the residue degree, shortest path closeness and betweenness. Betweenness centrality depicts the amount of times that a residue (node) acts as a bridge over the shortest paths between two nodes that are not directly connected. It measures the degree of significance of the bridge node and the weighted of connectivity between nodes in the global network. A node that continually appears in the shortest paths among other nodes (the shortest paths often contain that node) is more capable of facilitating communication among other nodes. The formula is as follows:

(1) $$\begin{array}{l} C_{B}\left(i\right)=\sum_{j<k}\frac{P_{jk}\left(i\right)}{P_{jk}} \end{array}$$

where *P*_*jk*_, determined by the Floyd–Warshall algorithm (Pallottino, 2010), is the number of shortest paths between nodes *j* and *k*. *P*_*jk*_*(i)* is the amount of shortest paths through node *i* between nodes *j* and *k* (Daly and Haahr, 2009).

Here, the Cytoscape plugin RINalyzer (Doncheva et al., 2011) was utilized to compute weighted centrality measures. For shortest paths centralities, the NrInteractions was chosen for attribution as the edge weight. The average weight option was performed to handle multiple edges, and negative weights were ignored in the network. Besides, the cutoff for weighted degree was set to 1 Å. The edge weights in RINs representing distances indicate that if the weight is smaller, the interaction is stronger. Therefore, it is imperative to convert them to distance scores by the max-value option.

### Free energy calculations

Molecular mechanics Generalized Born surface area (MM-GB/SA) methods (Hou et al., 2011, 2015; Sun et al., 2014a,b) implemented in Amber 14 package were applied to compute the binding free energy for the protein-ligand complex. For each system, the key coordinates were extracted from equilibrium trajectories in 10 ns after the MD simulations. In addition, the energy contribution of each residue to the protein and ligand interaction was analyzed through the decomposition of the binding free energy. In those methods, the binding free energy (Δ*G*_*bind*_) is deemed as the discrepancy of binding free energies among protein-ligand complex (*G*_*complex*_), the receptor (*G*_*protein*_) and ligand (*G*_*ligand*_), which is summarized as

(2) $$\begin{array}{l} \Delta G_{bind}=G_{complex}-(G_{receptor}+G_{ligand})=E_{MM}\\ \;+G_{sol}-T\Delta S \end{array}$$

(3) $$E_{MM}\;=\;E_{ele}\;+\;E_{vdw}$$

(4) $$G_{sol}=G_{\text{GB}}+G_{\text{SA}}$$

In the Equations (2) and (3), *E*_*MM*_ denotes the gas phase interaction that contains electrostatic (*E*_*ele*_) and van der Waals (*E*_*vdW*_). In the Equation (4), *G*_*sol*_ represents solvation energy which separates into an electrostatic/polar solvation free energy (*G*_*GB*_) and a nonpolar solvation free energy (*G*_*SA*_). *G*_*GB*_ is obtained from the Generalized Born (GB) method, and *G*_*SA*_ is acquired from the molecular solvent accessible surface area (SASA). Moreover, the -*T*Δ*S* value in the Equation (2) signifies the variation of the conformational entropy, which was evaluated from the 200 frames randomly collected from the equilibrium trajectories using the *nmode* program in AMBER 14.0 (Case et al., 2005). In our calculations, the GB model was calculated by GB-OBC1. The atomic radii were both set to the default bondi. The values of the polar solvation free energy (*G*_*GB*_) were set to default (ε = 1) and the nonpolar part (*G*_*SA*_) was calculated by the LCPO method: *G*_*SA*_ = 0.0072 × ΔSASA.

### Pathways identified with CAVER analyst 2.0

CAVER Analyst 2.0 is normally used to quantify pathways in proteins that approximate buried active sites, and to visualize static and dynamic structures in tunnels and channels in real time (Chovancova et al., 2012). At this point, the dynamic structures (1,000 snapshots) were originated from 10 ns equilibrium trajectores. In the tunnel computation setting, the starting point was set at the position of the ligand (D-fructose). The probe radius and the clustering threshold were set to 1 and 4.5 Å, correspondently. The shell depth and radius were set to 4 and 3 Å, accordingly. And other parameters used default settings throughout the calculations.

### Adaptive steered molecular dynamics (ASMD) simulations

ASMD simulations (Ozer et al., 2012; Bureau et al., 2016) were performed for wild-type and Y68I/G109P mutant complexes using Amber 14 package (Case et al., 2005). The predetermined reaction coordinate was divided into numerous smaller stages, with each one having multiple simulations performed parallelly. In those stages, a single trajectory was selected from simulations where the energy value was closest to the Jarzynski average (JA) (Jarzynski, 1997; Ozer et al., 2012). The swarm of trajectories were contracted into one single JA structure, which can help remove trajectories that may contribute least to the overall Potential Mean of Force (PMF). The PMF calculation can inspect the change of the energy of the system as a function of the geometrical coordinate (the distance between the ligand and the protein). In the ASMD simulations, the pulling direction was determined with the vector of two atoms in accordance with the top frequently observed pathway identified by CAVER Analyst 2.0. The stretching velocity was 10 Å/ns, and the spring constant k was 40 kcal/(mol × Å^2^). The ligand was pulled roughly along the reaction coordinate from 4 to 30 Å. The reaction coordinate could be split into 14 stages, with each one carrying out 24 simulations for each protein-ligand system. When the reaction coordinate reached 30 Å, the ligand was completely dissociated from binding sites of the protein.

## Results and discussions

### Structural validation of the modeled DPEase protein and structure-based sequence alignments

In our study, crystal structures of *Cc*DPEase (PDB ID: 3VNK) (Chan et al., 2012) and *Af* DPEase (PDB ID: 2HK1) (Kim et al., 2006) bound to D-fructose were chosen as multiple templates to the model. The modeled three-dimensional structure of *Cb*DPEase was selected on the grounds of the least DOPE score −36561.49. The plotted DOPE score profile for per residue was demonstrated in Figure S1, which mirrored the relatively similar energy between models and templates. The psi and phi distribution of the Ramachandran plot for the modeled *Cb*DPEase protein showed that 93.0% of residues were present in the most favorable regions, and 6.2% of residues were in the additional allowed regions. There were 0.4% of the residues in generously allowed regions and 0.4% of them in the disallowed region (Figure S2). The average overall quality factor evaluated by ERRAT2 program was 87.633%, which was within the expected range and ensures the model quality (Figure S3). The result of Verify3D indicated that 98.97% of residues had averaged 3D-1D score ≥ 0.2 (Figure S4). These validation studies concluded that bonded, non-bonded interactions and backbone conformations were within the limits of the reliable structure.

**Figure 1:**
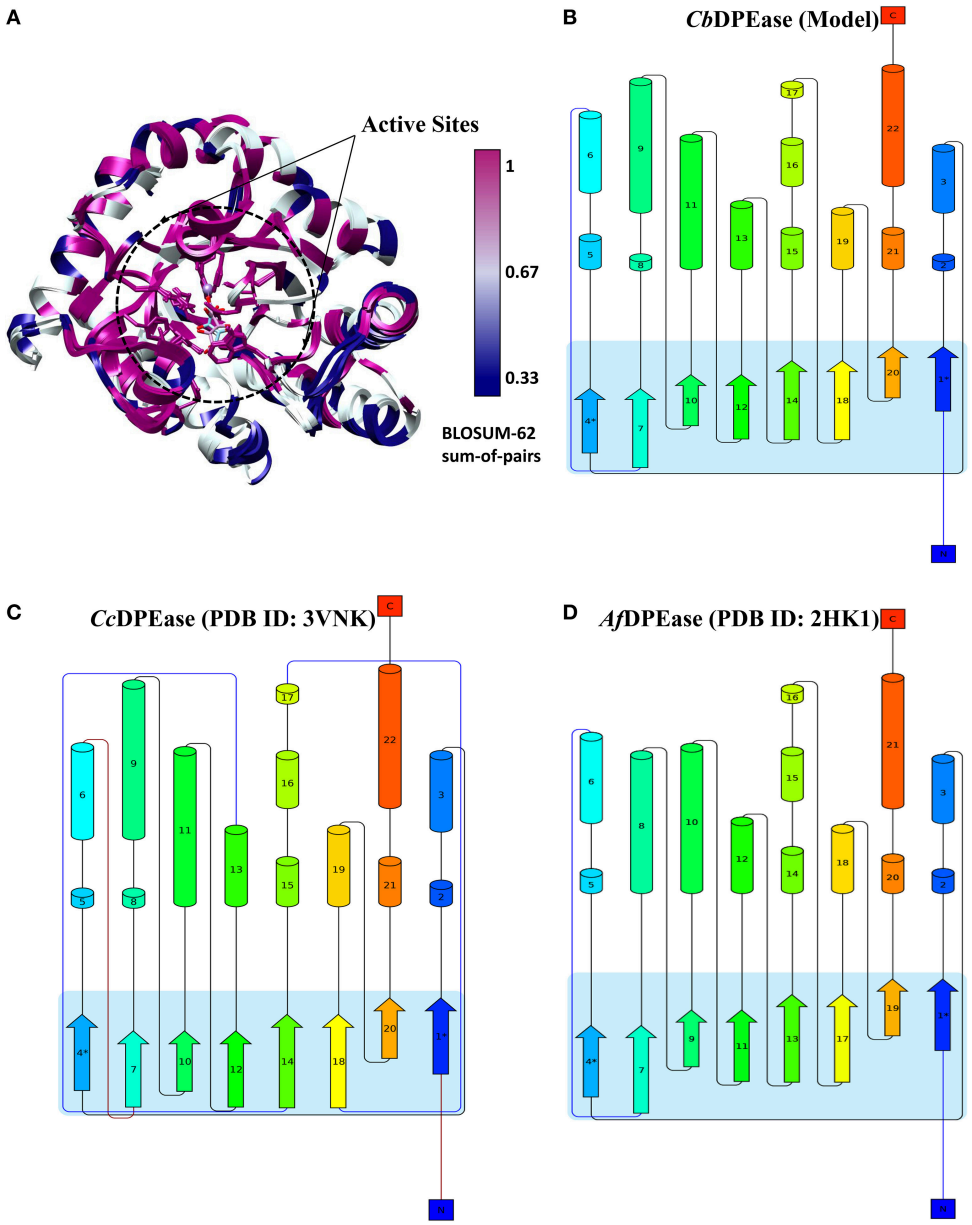
**(A)** The map of sequence conservation onto structures with Chimera. The secondary structure elements of **(B)** *Cb*DPEase (model), **(C)** *Cc*DPEase (template), **(D)** *Af* DPEase (template) are provided by Pro-origami.

To explore the structural similarity between crystal structures of DPEase and modeled structures, a multiple sequence alignment and two superimpositions of the targeted protein and templates were displayed in Figure S5 and Table 1. It showed that (1) the Root Mean Square Deviation (RMSD) of 0.278 Å was over 153 amino acid pairs for *Cc*DPEase and RMSD of 0.599 Å was over 136 amino acid pairs for *Af* DPEase. It also reflected that the similar sequence alignment scores (1030.5 and 967.1 respectively) were related to the two templates. Moreover, the sequence conservation was mapped onto the structure to compare the conserved regions of templates and the modeled protein. The map in Figure 1A showed that structures at the active sites were mostly conserved and associated with the most similar sequence (shown in purple). Then, the secondary structure elements visualized in Figures 1B–D gave us a clear depiction of high similarity of chains and the secondary structure elements for templates and the modeled protein. All the above results provided an ample evidence to demonstrate that the modeled protein structurally homologous to the *Cc*DPEase (PDB ID: 3VNK) and the *Af* DPEase (PDB ID: 2HK1), showing the reliability for further simulations.

**Table 1:** The results of Sequence alignment for *Clostridium cellulolyticum* DPEase (PDB ID 3VNK) and Model, *Agrobacterium fabrum* DPEase (PDB ID 2HK1) and Model generated with MatchMaker and Match-Align modules are visualized in UCSF Chimera.

**Reference structure**	**Match structure**	**N/RMSD**	**Sequence alignment score**
3VNK	Model	153/0.278	1030.5
2HK1	Model	136/0.599	967.1

### Integrative visual analyses for residue interactions networks of protein structures

**Figure 2:**
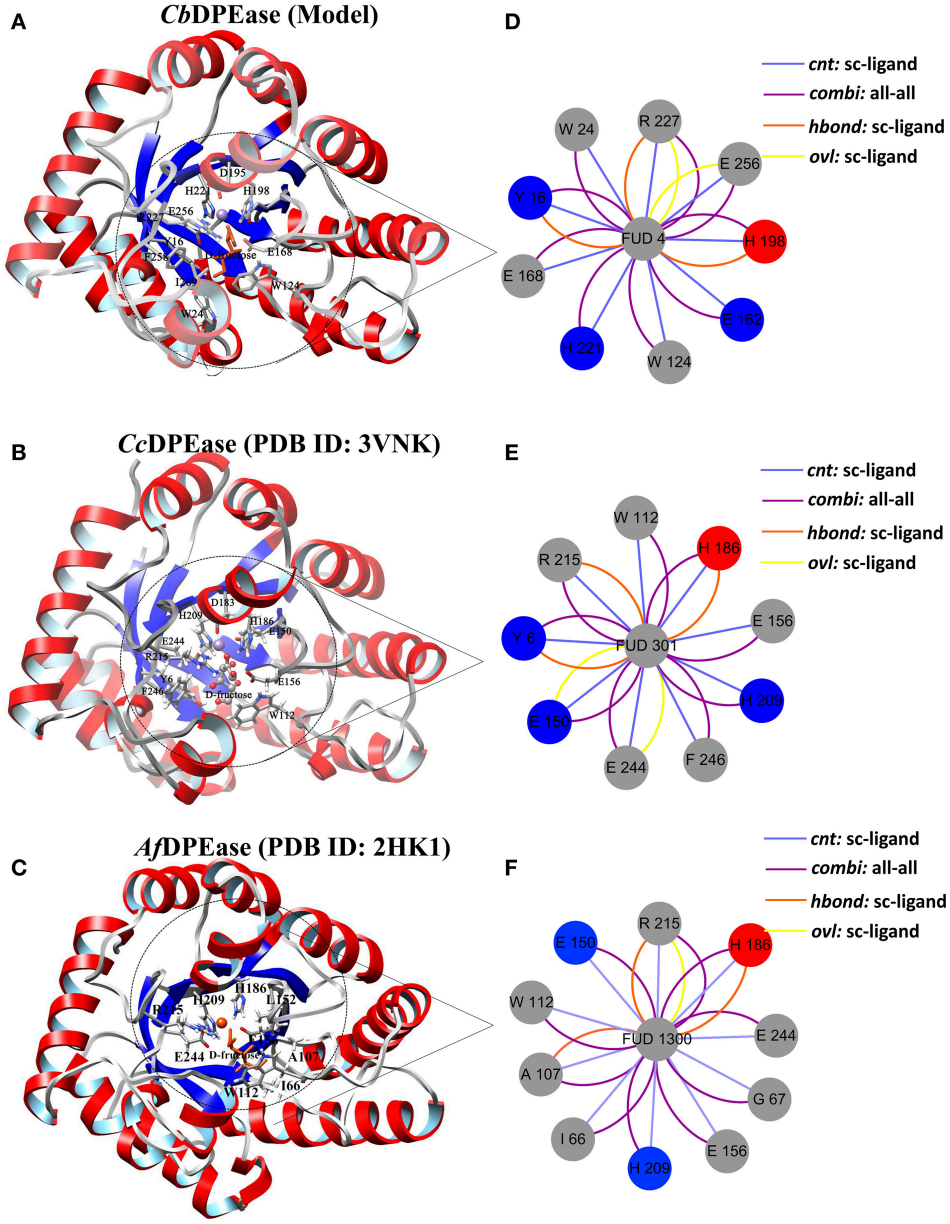
Simultaneous visualization of biological information for templates and model structures using different complementary views. Three-dimensional structures of **(A)** *Cb*DPEase (model), **(B)** *Cc*DPEase (template), and **(C)** *Af* DPEase (template) are shown with UCSF Chimera. Two-dimensional view of the subnetwork of the substrate (Fructose) for **(D)** *Cb*DPEase (model), **(E)** *Cc*DPEase (template), and **(F)** *Af* DPEase (template). The interaction types: *cnt (interatomic contact):* sc-ligand colored by light blue, *combi* (*combined):* all-all colored by purple, *hbond:* mc-ligand colored by red, *hbond:* sc-ligand colored by orange, *ovl (overlap):* sc-ligand colored by yellow.

The optimized structure of D-fructose was docked in the model of *Cb*DPEase. Superimposition of molecular docking conformation (blue) and crystal structure (red) was demonstrated in Figure S6, which indicated that the substrate was properly positioned in the active site. To survey the rationality of the docking from the perspective of the interaction between the substrate and the protein, we extracted subnetworks of the substrates of *Cb*DPEase, *Cc*DPEase, and *Af* DPEase (Figure 2). It was evident that the active residues around the D-fructose for the three structures were literally alike and conservative, such as W112 in *Cc*DPEase and W112 in *Af* DPEase corresponding to W124 in *Cb*DPEase (W112_*Cc*DPEase_/W112_*Af*DPEase_/W124_*Cb*DPEase_), E150_*Cc*DPEase_/E150_*Af*DPEase_/E162_*Cb*DPEase_,E156_*Cc*DPEase_/E156_*Af*DPEase_/E168_*Cb*DPEase_,H186_*Cc*DPEase_/E186_*Af*DPEase_/H198_*Cb*DPEase_,H209_*Cc*DPEase_/E209_*Af*DPEase_/H221_*Cb*DPEase_,R215_*Cc*DPEase_/E215_*Af*DPEase_/R227_*Cb*DPEase_,and E244_*Cc*DPEase_/E244_*Af*DPEase_/E256_*Cb*DPEase._ Hence, the docking complex for *Cb*DPEase could be used to the next simulations.

### Representative structures of the wild-type and Y68I/G109P mutant

The Free Energy Landscape (FEL) map shown in Figures S8A–D was devoted to characterizing the first two principal components of motion in equilibration stage simulations. From those analyses, the lowest-energy structures of the wild-type *Cb*DPEase and Y68I/G109P mutant from the first two equilibrium trajectories were achieved after 9.3 and 9.9 ns respectively (Figures S8A,B). Similarly, based on the latter two equilibrium trajectories of the wild-type *Cb*DPEase and Y68I/G109P mutant (see Materials and Methods), the structures located in the lowest-energy region of FEL, 7.6 and 6.9 ns, were considered as representative structures (Figures S8C,D). Consequently, all these structures were selected as representative structures and used for the next calculations (network analysis and ASMD simulations).

### Structure-activity relationship of the *Cb*DPEase and Y68I/G109P mutant: network centrality analysis

**Figure 3:**
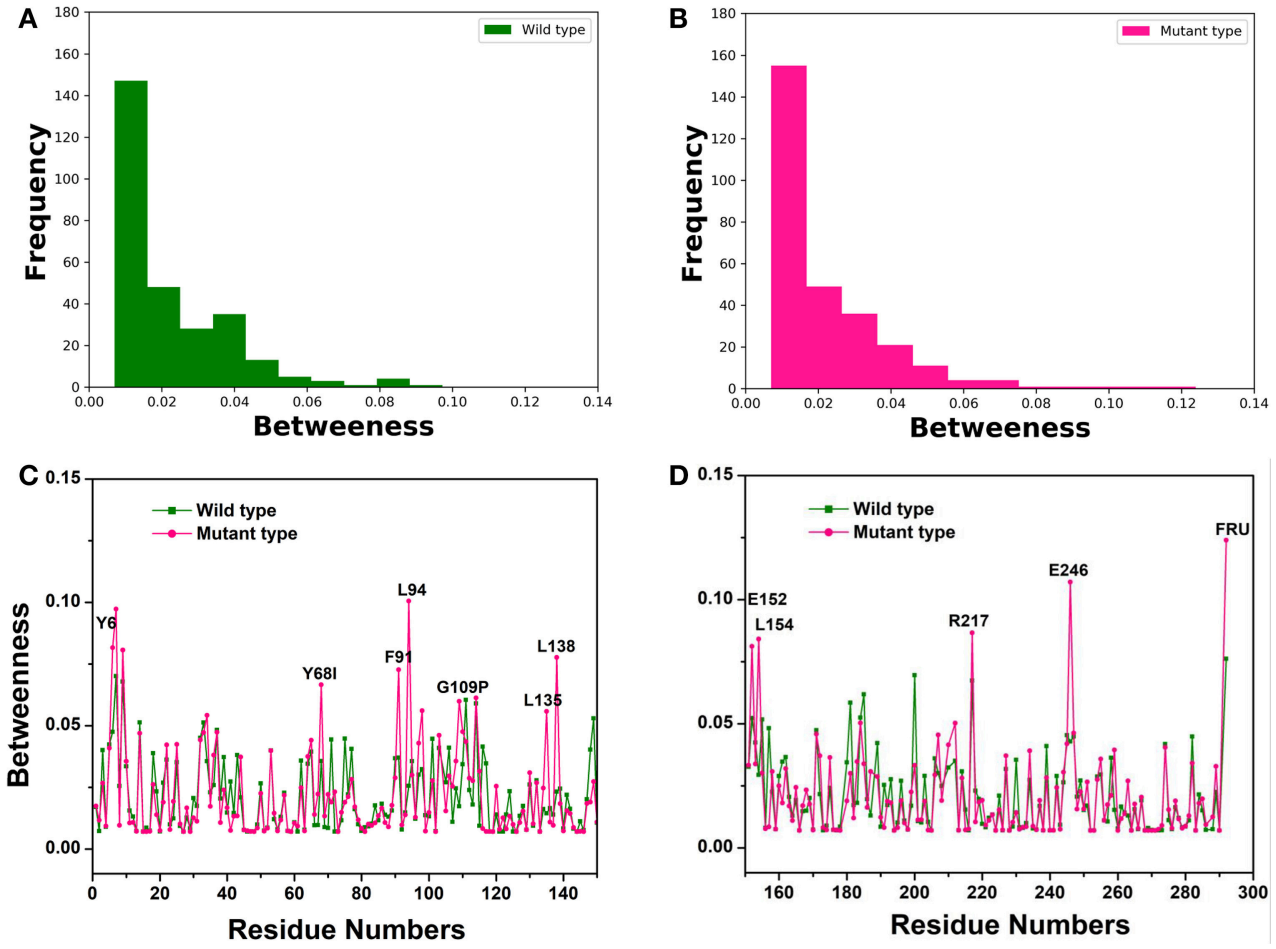
The Network centrality analysis of the representative structures from the first two equilibrium trajectories. The frequency distributions of the betweenness values of the **(A)** wild-type structure (green bars), **(B)** Y68I/G109P mutant type structure (pink bars). Residue-based betweenness profiles of the **(C)**, wild-type structure (green) and **(D)** Y68I/G109P mutant type structure (pink). The residue-based betweenness distributions point to small-world organization of the interaction networks in the *Cb*DPEase structures. The distributions of protein structure network parameters are obtained by the representative structures in equilibration stage.

To recognize crucial mediating nodes (residues) involved in network graphs for the representative structures from the first two equilibrium trajectories, frequency distributions of the betweenness values were identified as shown in Figures 3A,B. It indicated the frequency of nodes lying on paths of other nodes in the networks graphs. The betweenness profile in the Y68I/G109P mutant manifested a greater distribution in low betweenness value (0.01~0.02), which was a sharper decay and a longer tail of the distribution in comparison with the wild-type *Cb*DPEase. Thus, mutations at Y68 and G109 may trigger a few functional residues to act as significant bridges on the communication between other nodes to promote the catalytic activity of the *Cb*DPEase. To confirm this conjecture, the network centrality for every mediating residue was examined to track the relationship between centrality and functional activities of key residues in the wild-type *Cb*DPEase and Y68I/G109P mutant. Unlike the wild-type *Cb*DPEase, the betweenness in the Y68I/G109P mutant revealed the noticeable peaks in keeping with the functional residues in the active pocket (Y6, E152, L154, R217, and E246), mutated residues (Y68I, G109P) and certain neighboring residues nearby the active sites (F91, L94, L135, L138) (Figures 3C,D). Furthermore, highly similar results were also occurred in the betweenness centrality of the representative structures from the latter two equilibrium trajectories (Figure S9). Based on the proposed catalytic mechanism and substrates binding studies of *Cc*DPEase bound to D-fructose, the residues mentioned as follows are affordable for the catalytic activity of *Cb*DPEase, E244_*Cc*DPEase_/E246_*Cb*DPEase_, which are coordinated with the Co^2+^. Firstly, it removes a proton from C3, and then E150_*Cc*DPEase_/E152_*Cb*DPEase_ protonates C3 on the opposite side (Chan et al., 2012). And Y6_*Cc*DPEase_/Y6_*Cb*DPEase_, E150_*Cc*DPEase_/E152_*Cb*DPEase_, R215_*Cc*DPEase_/R217_*Cb*DPEase_, and E244_*Cc*DPEase_ /E246_*Cb*DPEase_ can make hydrogen bonds with D-fructose (Chan et al., 2012). Moreover, the Y68I/G109P mutant enzyme is more thermostable and it demonstrates outstanding catalytic ability, which is 1.7-fold for t_1/2_ value and 1.2-fold for k_cat_/K_m_ of the wild-type *Cb*DPEase correspondently (Zhang et al., 2016).

Overall, these results of network centrality analysis suggested that catalytic activities of the wild-type *Cb*DPEase and Y68I/G109P mutant could be mediated by residues in the active pockets. Interestingly, these functional residues could be surrounded and shielded by neighboring residues with high betweenness values, which could enhance the communications of nodes in networks. The betweenness analysis of the Y68I/G109P mutant detected noticeable peaks in several functional residues, which showed functional residues to be more capable of communicating with neighboring nodes in comparison with the wild-type *Cb*DPEase. Thus, site-directed mutagenesis of Y68 and G109 could enhance the substrate-binding affinity and catalytic efficiency of *Cb*DPEase, which was consistent with experiments (Zhang et al., 2016).

### Small-world interaction networks analysis

To detect the close interaction cooperativity from the representative structures of the first two equilibrium trajectories, we gradually measured the small-world interaction networks mediated by functional residues in the following passages.

**Figure 4:**
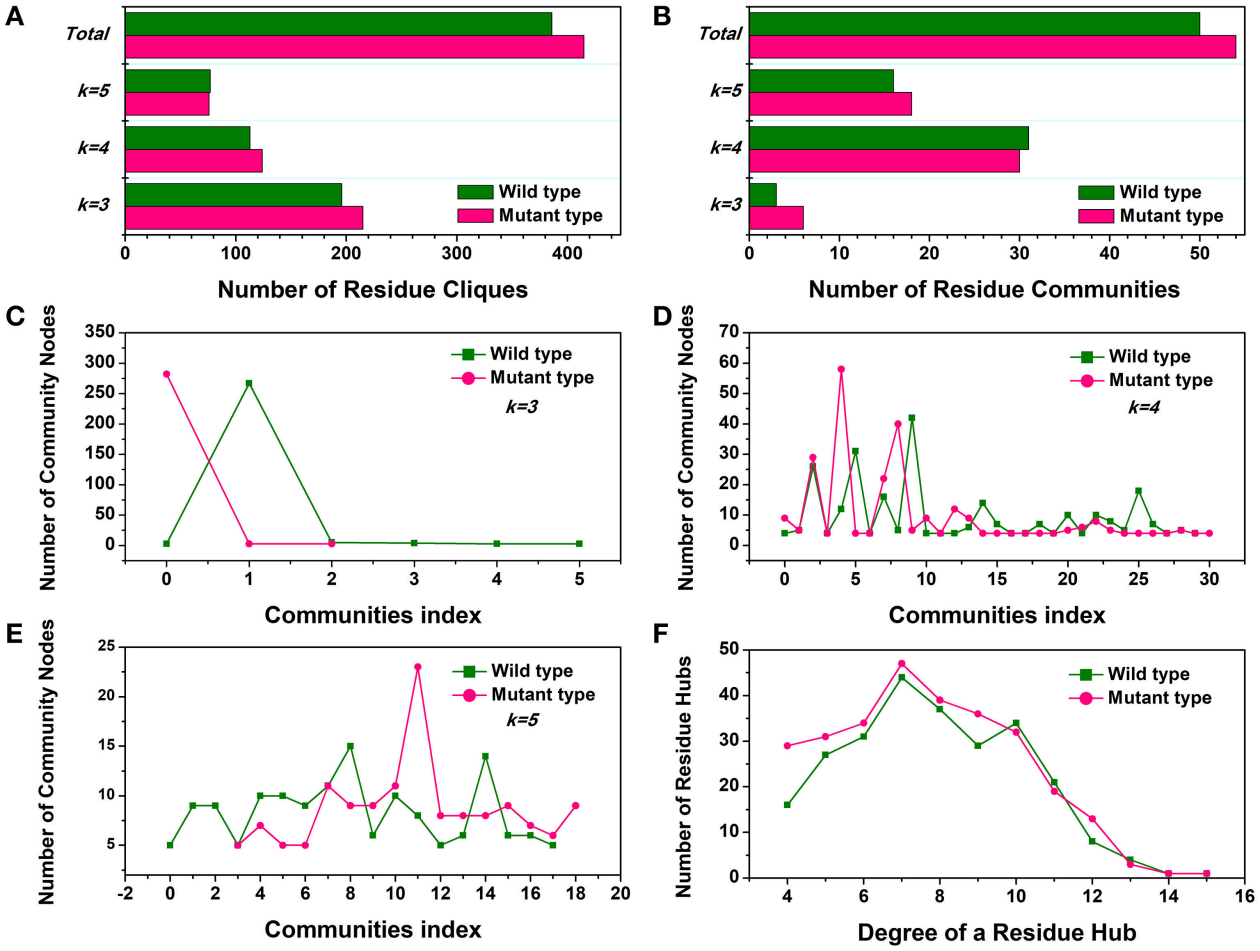
The small-world interaction networks analysis of the *Cb*DPEase (model) structures. **(A)** The distribution of cliques in the wild-type structure (green) and the Y68I/G109P mutant type structure (pink). **(B)** The distribution of communities in the wild-type structure (green) and the Y68I/G109P mutant type structure (pink). The number of community nodes for **(C)**, *k* = 3, **(D)** *k* = 4, **(E)** *k* = 5 in the wild-type structure (green) and the Y68I/G109P mutant type structure (pink). **(F)** The degree distribution of residue hubs in the wild-type structure (green) and the Y68I/G109P mutant type structure (pink). The distributions of network parameters are obtained by the representative structures in equilibration stage.

First, the number of cliques and communities were computed to characterize the stable module of interconnected residues and mutagenesis-induced variations in small-world interaction networks. The mutant cliques graph displayed a greater number of local cliques (Cliques number = 415) than the wild-type (Cliques number = 386) (Figure 4A). The more densely interconnected cliques could establish much steadier communities in the Y68I/G109P mutant. The analysis of community (Figure 4B) indicated that the total amounts of stable communities in the Y68I/G109P mutant were larger than the wild-type *Cb*DPEase. Interestingly enough, the numbers of interconnecting residues (nodes) in certain communities consisting of different cliques (*k* = 3, *k* = 4, *k* = 5), compared to wild-type, were distinctly high in Y68I/G109P mutant (portrayed as pink peaks) (Figures 4C–E), which indicated the higher cooperativity in the mutant type.

Second, the distribution of local residue hubs in the conformational networks was also characterized and shown in Figure 4F. The hubs were highly connected nodes (at least 4) in the network. The result showed that the number of hub nodes increased markedly when the degree of the residue hub was low. Then, the exponentially decayed, as the degree of the residue hub increased in the wild-type *Cb*DPEase and Y68I/G109P mutant, which was in agreement with previous network-based studies of proteins (Atilgan et al., 2003; del Sol et al., 2005). More interestingly, the larger distribution of residue hubs detected in the Y68I/G109P mutant could be consistent with the tighter cooperativity of communities in the mutant protein, which may be the crucial evidence of increased activities of *Cb*DPEase.

**Figure 5:**
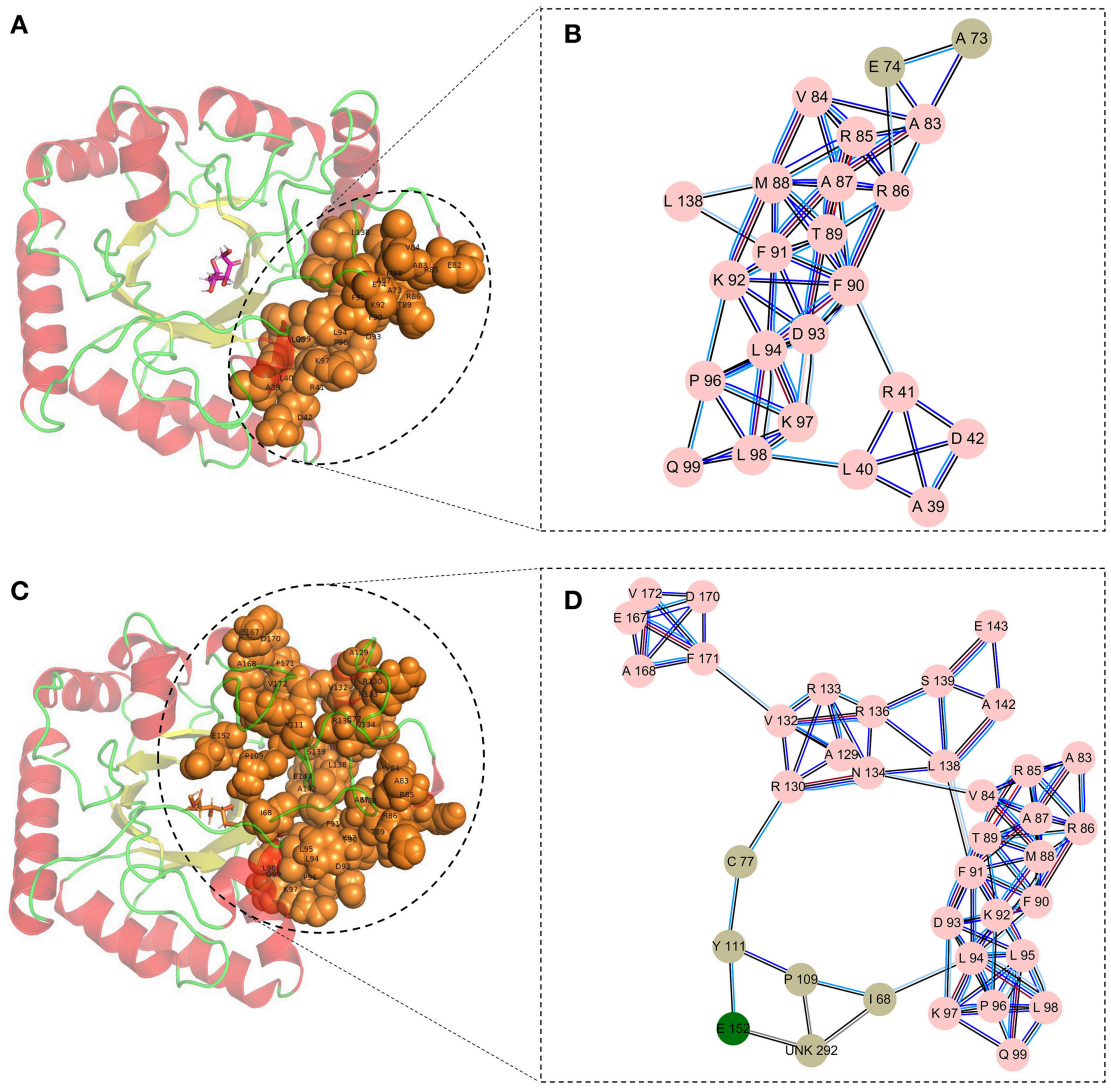
The cluster network analysis. **(A)** General overview and **(B)** a detailed close-up of the network of cluster in the wild-type structure. **(C)** General overview and **(D)** a detailed close-up of the network of cluster in the Y68I/G109P mutant type structure.

Finally, the cluster network analysis was displayed as a detailed map of the residue interaction network that could detect densely interconnected modules in local networks. The map of the cluster analysis suggested that compared with the wild-type *Cb*DPEase (Figures 5A,B), the highly interconnected nodes included the substrate, the functional residues (E150_*Cc*DPEase_/E152_*Cb*DPEase_), and the mutant sites (I68 and P109) that could form a larger and denser cluster in the Y68I/G109P mutant (Figures 5C,D).

It worth to noting that through the network analysis of the representative structures from the latter two equilibrium trajectories (Figures S10, S11), we found that for the wild-type *Cb*DPEase and Y68 and G109 mutant, the differences in the number of cliques and communities, the trend of the hubs, and the intensities of the clusters were highly compatible with the above conclusions. Overall, site-directed mutagenesis of Y68 and G109 of the *Cb*DPEase may induce more stable interactions than that of the wild-type *Cb*DPEase in the local network, which corresponded to the high level of cooperativity that may affect the catalytic activity of *Cb*DPEase.

### Enhancement of protein-ligand interactions with site-directed mutagenesis of Y68 and G109

Reliable free energy calculations based on the first two equilibrium trajectories were performed to assess the quantitative effects of the binding affinities between the protein and the ligand. As can be seen in Table 2, Δ*G*_*bind*_ of D-fructose bound to the Y68I/G109P mutant was −13.23 kcal/mol, which was nearly 11 kcal/mol lower than that of wild-type complex (−2.49 kcal/mol). It meant that the binding affinities of the ligand in the Y68I/G109P mutant were stronger than that in the wild-type *Cb*DPEase, which were in accord with the experiment results (the *k*_*cat*_*/K*_*m*_ in the Y68I/G109P mutant increased by 1.2-fold compared with the wild-type) (Zhang et al., 2016). And the binding free energies were driven by the electrostatic interaction (Δ*E*_*ele*_), polar solvation (Δ*G*_*GB*_), the *vdW* interaction (Δ*E*_*vdW*_), nonpolar solvation (Δ*G*_*SA*_), and entropy (–TΔ*S*). To be specific, the contributions of Δ*E*_*ele*_ to the binding energies varied substantially in the wild-type *Cb*DPEase (−59.39 kcal/mol) and Y68I/G109P mutant (−72.82 kcal/mol), which were favorable to binding. And the value of conformational entropy (–TΔ*S*) reached 19.95 kcal/mol and 19.46 kcal/mol in the wild-type *Cb*DPEase and Y68I/G109P mutant respectively, which were always unfavorable to binding. Moreover, there was almost no considerable difference in the Δ*E*_*vdW*_, Δ*G*_*GB*_, and Δ*G*_*SA*_ between the wild-type *Cb*DPEase and Y68I/G109P mutant. This showed that the *vdW* energy, polar solvation energy, and nonpolar solvation energy did not significantly contribute to the disparity of binding free energy. The free energy contributions were decomposed to the binding free energy in individual residues (Figure S12), which indicated that contributions in E152, E264, D185, and H211 of the Y68I/G109P mutant were far lower than that of the wild-type *Cb*DPEase corresponding the active site in *Cb*DPEase.

**Table 2:** Energy terms obtained using MM-GB/SA for the D-fructose bound to the wild-type *Cb*DPEase and Y68I/G109P mutant (kcal/mol).

**System**	**Wild-type *Cb*DPEase**	**Y68I/G109P mutant**
*ΔE_*vdW*_*	−16.67 ± 3.95	−15.61 ± 3.37
*ΔE_*ele*_*	−59.39 ± 10.31	−72.82 ± 7.31
*ΔG_*GB*_*	57.42 ± 4.19	59.62 ± 4.81
*ΔG_*SA*_*	−3.81 ± 0.14	−3.88 ± 0.15
*ΔG_*polar*_*^a^	−1.97	−13.20
*ΔG_*nonpolar*_*^b^	−20.48	−19.49
*ΔG_*MM*−*GB*/*SA*_*^c^	−22.44 ± 5.52	−32.69 ± 3.31
*-TΔS*	19.95 ± 5.37	19.46 ± 4.95
*ΔG_*bind*_*^d^	−2.49	−13.23
*k_*cat*_/K_*m*_* (min^−1^ mM^−1^)^e^	59.2 ± 2.9	73.7 ± 6.2

a ΔG_polar_ = ΔE_ele_+ΔG_GB_

b ΔG_nonpolar_ = ΔE_vdW_ +ΔG_SA_

c ΔG_MM−GB/SA_ = ΔE_ele_+ΔG_GB_ +ΔE_vdW_ +ΔG_SA_

d ΔG_bind__=_ ΔG_MM−GB/SA_ − TΔS

e *k_cat_/K_m_ values obtained from the experimental data (min^−1^ mM^−1^) (Zhang et al., 2016)*.

Furthermore, we applied the second free energy calculation for snapshot structures extracted from the latter two equilibrium trajectories, the results in Table S1 indicated that the binding free energy of the Y68I/G109P mutant lower than that of the wild-type *Cb*DPEase. And the contributions of non-polar part, polar part, and conformational entropy to the binding free energies were in line with previous results. Conclusion could be drawn from those results that the combination of the D-fructose and the Y68I/G109P mutant was more stable than that of the wild-type *Cb*DPEase.

### Identification of pathways in equilibration trajectories

**Figure 6:**
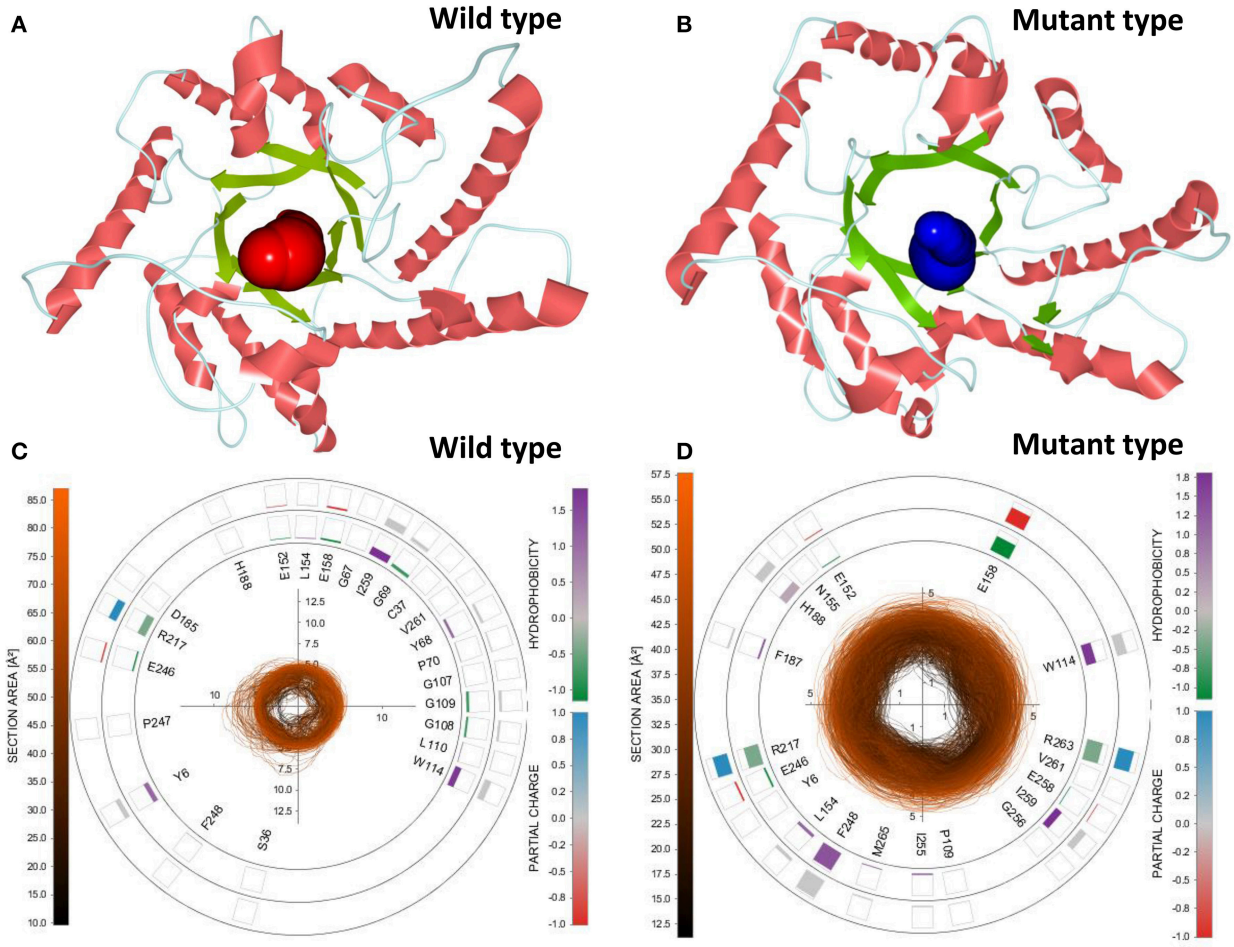
3D visualization of the two candidate tunnels (tunnel 1) of the **(A)** wild-type *Cb*DPEase and **(B)** Y68I/G109P mutant. The detailed exploration of the tunnel bottleneck contour (collar) over time for the **(C)** wild-type *Cb*DPEase, and **(D)** Y68I/G109P mutant.

To select the best tunnel candidates for ASMD simulations and identify the discrepancy in representational tunnels between the wild-type *Cb*DPEase and Y68I/G109P mutant, Caver Analyst 2.0 was utilized for the analysis of 1,500 snapshots from equilibration trajectories after MD simulations. All Tunnels Heat Maps (ATH) (Byška et al., 2015), representing the whole tunnels in the molecular dynamics, showed that a tunnel with most frequent occurrence of the Y68I/G109P mutant was detected in tunnel 1 (Figure S13B) with the larger bottleneck (the color encodes the bottleneck size) compared with that of the wild-type *Cb*DPEase (Figure S13A). Single Tunnel Heat Maps (STH) (Byška et al., 2015) in Figures S13C,D represented the most accessible tunnels (tunnel 1) of the wild-type *Cb*DPEase and Y68I/G109P mutant over time, incorporating the individual rectangles colored by the width of the tunnel. It indicated that tunnel 1 in the Y68I/G109P mutant was longer and wider than the wild-type *Cb*DPEase. The results of ATH and STH manifested that the best tunnel candidate selected in the Y68I/G109P mutant (tunnel 1) may facilitate the unbinding of D-fructose more easily than that in the wild-type *Cb*DPEase. After that, the 3D visualization of the two candidate tunnels (tunnel 1) of the wild-type *Cb*DPEase and Y68I/G109P mutant were shown in Figures 6A,B, and the detailed exploration of the tunnel bottleneck and surrounding amino acids over time were presented in 2D collars (Figures 6C,D). From the collars, the shape and the area of the bottleneck of the main tunnel in the Y68I/G109P mutant was more stable and wider than that of the wild-type *Cb*DPEase. The surrounding amino acids displayed around the contour demonstrated the frequency they participated in the tunnel boundary in the light of the hydrophobicity and the partial charge of atoms. It was observed that W114, E158, R217, and R263 in the Y68I/G109P mutant had a bigger impact on the tunnel than that in the wild-type *Cb*DPEase, which enabled the substrate of the outside solvent to easily pass through the tunnel into the buried active pocket of the Y68I/G109P mutant.

### Analysis of unbinding pathways of the wild-type *Cb*DPEase and Y68i/G109p mutant by ASMD simulation and PMF calculations

**Figure 7:**
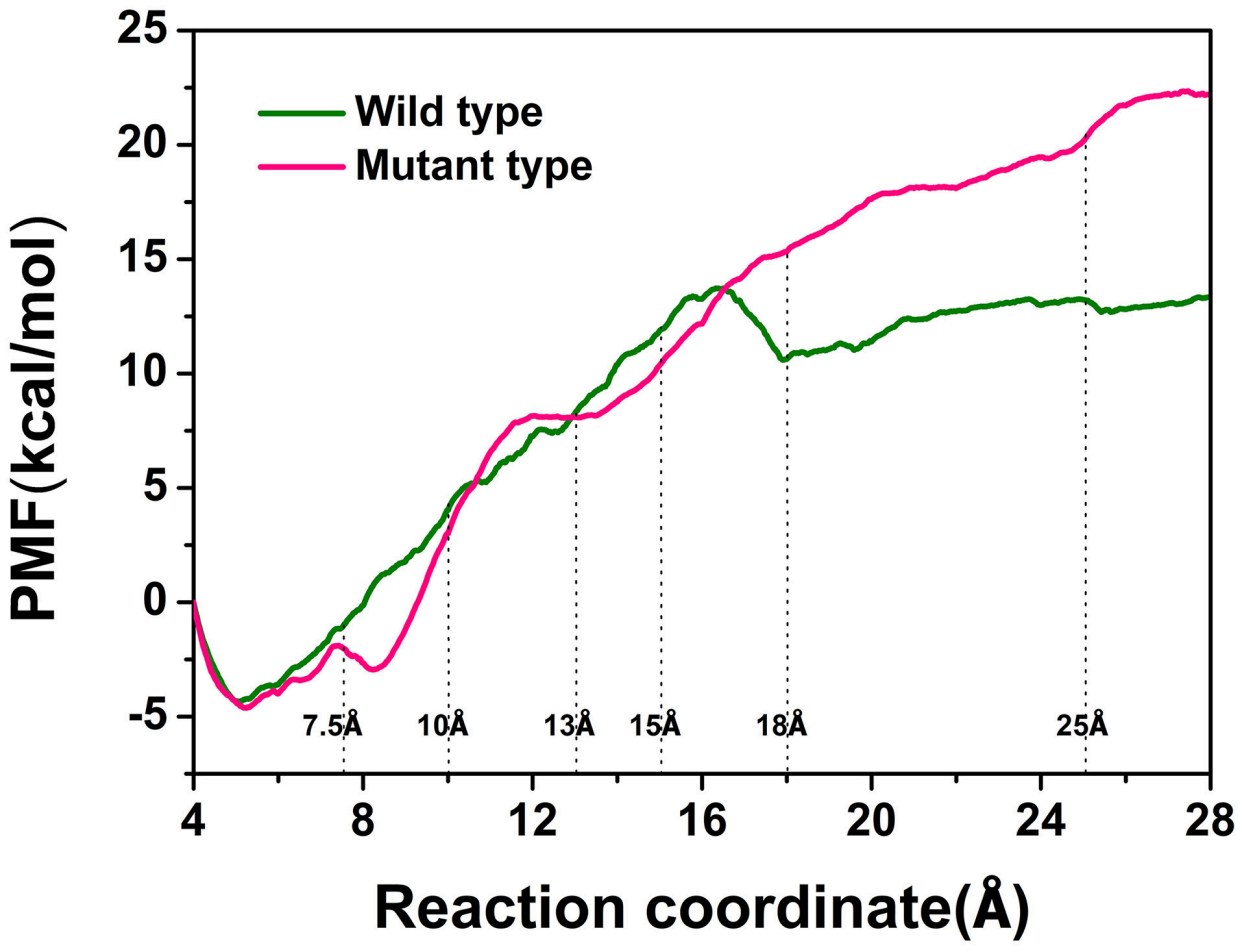
PMF profiles along the reactions coordinate for wild-type (green) and Y68I/G109P mutant type (pink).

To explore the protein-ligand interactions and the affinity of the active sites for the D-fructose during the dissociation of ligand in the wild-type *Cb*DPEase and Y68I/G109P mutant, we conducted ASMD simulations on the wild-type *Cb*DPEase and Y68I/G109P mutant bound to D-fructose. Here, PMF profile displayed the energy changes for the departure of the D-fructose from the wild-type *Cb*DPEase and Y68I/G109P mutant, which amounted to approximately 13 and 22 kcal mol^−1^ respectively (Figure 7). In the Y68I/G109P mutant, the higher energy barrier should be transferred to totally dissociate of D-fructose from the tunnel. In other words, the D-fructose had stronger binding interactions with the Y68I/G109P mutant than wild-type *Cb*DPEase.

**Figure 8:**
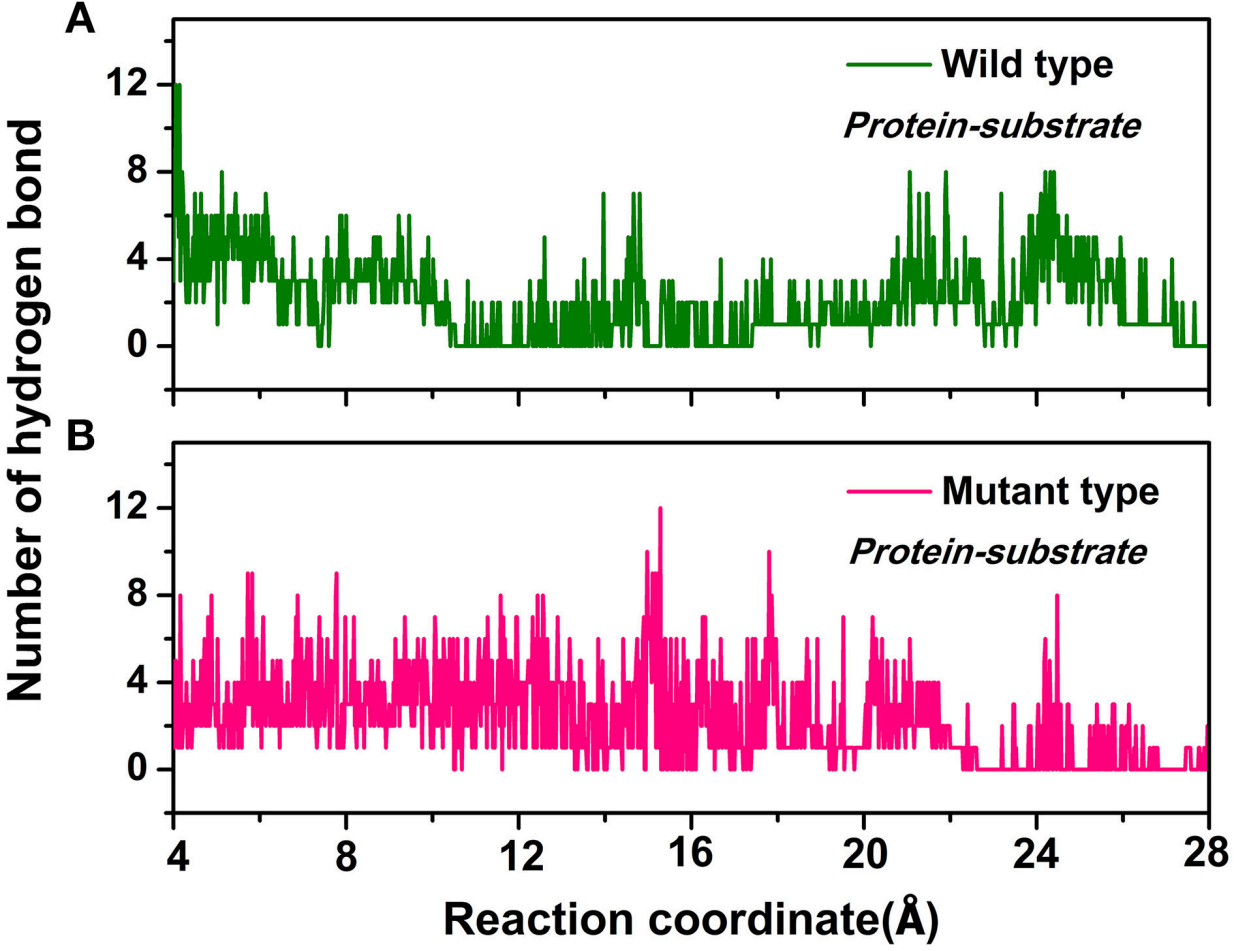
The number of hydrogen bonds change between D-fructose and tunnel residues along the reaction coordinate in the **(A)** wild-type *Cb*DPEase and **(B)** Y68I/G109P mutant.

Furthermore, the hydrogen bond interactions between the protein and the ligand were detected. It assisted in the understanding of the dissociation pathways and provides some vital clues for improving the catalytic efficiency by the site-directed mutagenesis of Y68 and G109. As shown in Table 3, in the Y68I/G109P mutant, the likelihood of hydrogen bond between D-fructose and tunnel residues was higher than that of the wild-type. Especially in the Y68I/G109P mutant, E246 coordinated with the Co^2+^ could make stronger hydrogen bond interactions with D-fructose, which may help a proton divert from C3 in D-fructose (Chan et al., 2012). Hereafter, certain hydrophobic residues (e.g., Y6, W114) and polar residues (e.g., E34 and E158) developed previously as crucial active sites for the substrate binding (Chan et al., 2012) were more likely to form hydrogen bond interactions with D-fructose of the Y68I/G109P mutant. During the dissociation, the number of hydrogen bonds that changed between D-fructose and tunnel residues along the reaction coordinate were shown in Figure 8. Interestingly, there was a sudden increase in the amount of hydrogen bonds in the Y68I/G109P mutant after 14 Å along the reaction coordinate, resulting in the sharp increase of free energy value. Thereafter, the number of hydrogen bonds continued to be more than that of the wild-type *Cb*DPEase. Hence, the stronger protein-substrate interactions (hydrogen bond) in the Y68I/G109P mutant may facilitate the approach of the substrate (D-fructose) to the active pocket and induce a more efficacious catalytic reaction.

**Table 3:** The occupancies of hbond interaction occurred between protein and substrate (Fructose) for the Wild-type and the Y68I/G109P Mutant type.

**System**	**Donor**	**Acceptor**	**Probability%**
WT *Cb*DPEase	FRU: O3	GLU158: OE2	18.17
	FRU: O1	GLU152: OE2	7.67
	FRU: O1	GLU158: OE2	5.17
	FRU: O1	GLU158: OE1	5.17
	FRU: O4	GLU158: OE2	5.58
	HIS188: NE2	FRU: O2	5.67
	FRU: O6	GLU34: OE1	16.33
	GLY109: N	FRU: O2	8.08
Y68I/G109P *Cb*DPEase	FRU: O6	GLU158: OE2	20.92
	FRU: O5	GLU158: OE2	17.83
	FRU: O5	GLU158: OE1	11.33
	FRU: O1	GLU158: OE1	5.67
	FRU: O4	GLU34: OE1	12.25
	FRU: O1	GLU34: OE2	11.50
	FRU: O3	GLU34: OE1	7.83
	FRU: O1	GLU246: OE2	6.00
	FRU: O5	ILE68: O	7.83
	TYR6: OH	FRU: O6	9.67
	FRU: O6	TYR6: OH	9.67
	TRP114: NE1	FRU: O3	8.92
	FRU: O3	TRP114: NE1	8.92
	FRU: O4	TYR157: O	7.17
	FRU: O3	GLU152: OE2	5.17

**Figure 9:**
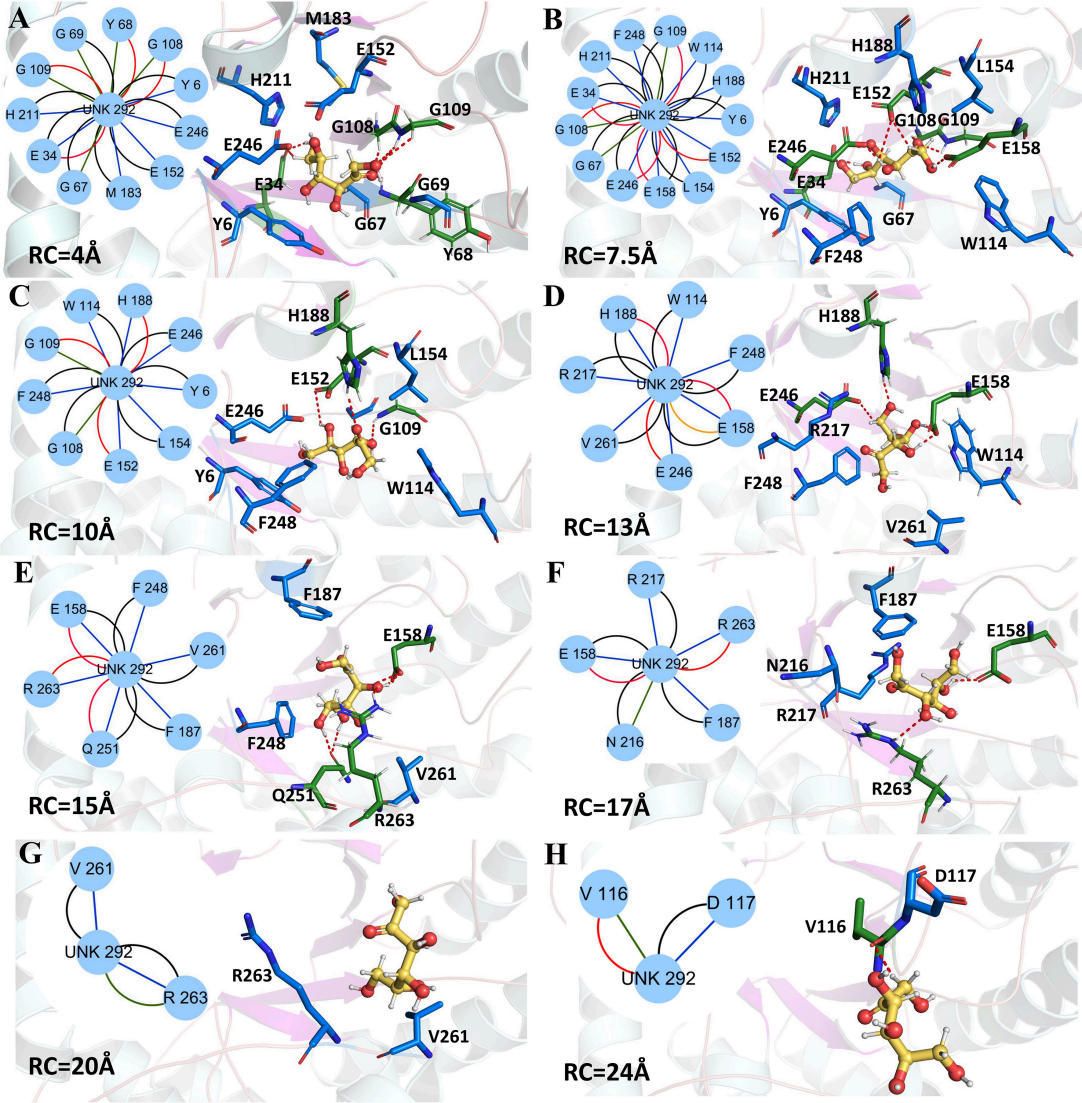
Representative structures in the dissociation process of the D-fructose from the wild-type *Cb*DPEase during ASMD simulations. The detailed location of **(A–H)**, along the dissociation process at 4, 7.5, 10, 13, 15, 17, 20, and 24 Å, respectively. The residues forming hydrogen bonds (red dashed line) in ligand dissociation are highlighted in green. The residues participating in the other interactions are highlighted in blue. The D-fructose is highlighted in yellow. Subnetwork analyses of the D-fructose are displayed as two-dimensional view. Possible interactions type occurred in ligand dissociation are interatomic contact (blue/green), hydrogen bond (red), overlap (yellow), and combined (black).

**Figure 10:**
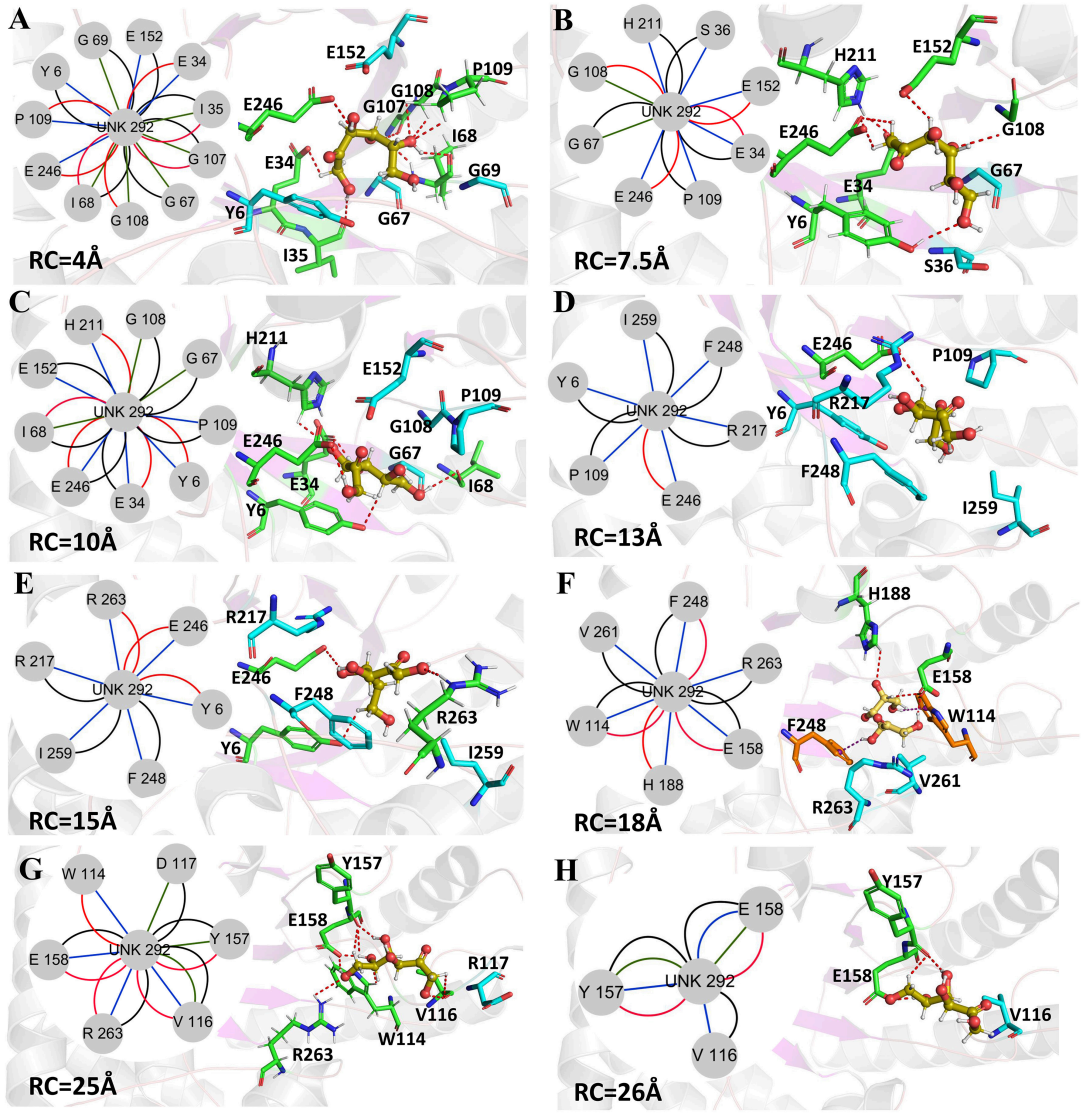
Representative structures in the dissociation process of the D-fructose from the Y68I/G109P mutant during ASMD simulations. The detailed location of **(A–H)**, along the dissociation process at 4, 7.5, 10, 13, 15, 18, 25, and 26Å, respectively. The residues forming hydrogen bonds (red dashed line) in ligand dissociation are highlighted in green. The residues forming Donor-π interactions (purple dashed line) in ligand dissociation are highlighted in orange. The residues participating in the other interactions are highlighted in cyan. The D-fructose is highlighted in yellow. Subnetwork analyses of the D-fructose are displayed as two-dimensional view. Possible interactions type occurred in ligand dissociation are interatomic contact (blue/green), hydrogen bond (red), overlap (yellow), and combined (black).

Now, the protein-ligand interactions for the wild-type *Cb*DPEase and Y68I/G109P mutant along the unbinding pathways were generated from protein subnetworks and displayed in the protein structure (Figures 9, 10). Possible interactions type occurred in the subnetwork were interatomic contact (blue/green) *(cnt)*, hydrogen bonds (red) *(hbond)*, the overlap (yellow) *(ovl)*, and the combined (black) *(combi)*.

The detailed analysis along the reaction coordinate for the unbinding pathway of the wild-type *Cb*DPEase was displayed in Figure 9. First, for the initial coordinate (RC = 4 Å), the representative subnetwork generated from the wild-type *Cb*DPEase (Figure 9A) showed that D-fructose was tightly fixed by hydrogen bond interactions with E34, Y68, G108, and G109. Also, it was fastened by functional residues (Y6, E34, G67, Y68, G69, G108, G109, E152, M183, H211, and E246) through the interatomic contact and combined interactions. Then at 7.5 Å, free energy value was experiencing a sharp increase due to the stronger hydrogen bond interactions between the D-fructose and the residues in active pockets (E34, G108, G109, E152, E158, and E246) than at 4 Å stage (Figure 9B). With the movement of the D-fructose, at 10~16 Å, relatively stable hydrogen bond interactions resulted in a steady increase in the free energy. Nevertheless, the residues that formed hydrogen bonds with the substrate were constantly varying. For example, residues G109, E152 and H188 at 10 Å (Figure 9C), residues H188, E158, and E246 at 13 Å (Figure 9D), and residues E158, Q251, and R263 at 15 Å (Figure 9E) all altered frequently. Thereafter, the interconnections including the hydrogen bonds between tunnel residues and D-fructose declined rapidly after ~16 Å (Figures 9F–H), giving rise to the sharp decrease in free energy value. It was worth noting that E158, as an important tunnel residue, could continue to generate the hydrogen bond with the D-fructose during 13–17 Å, which was line with the tunnel analysis results and the hydrogen bond probability. Finally, at ~28 Å, the D-fructose completely departed from the unbinding pathways of the wild-type *Cb*DPEase and the curves of PMF tended to be flat.

**Figure 11:**
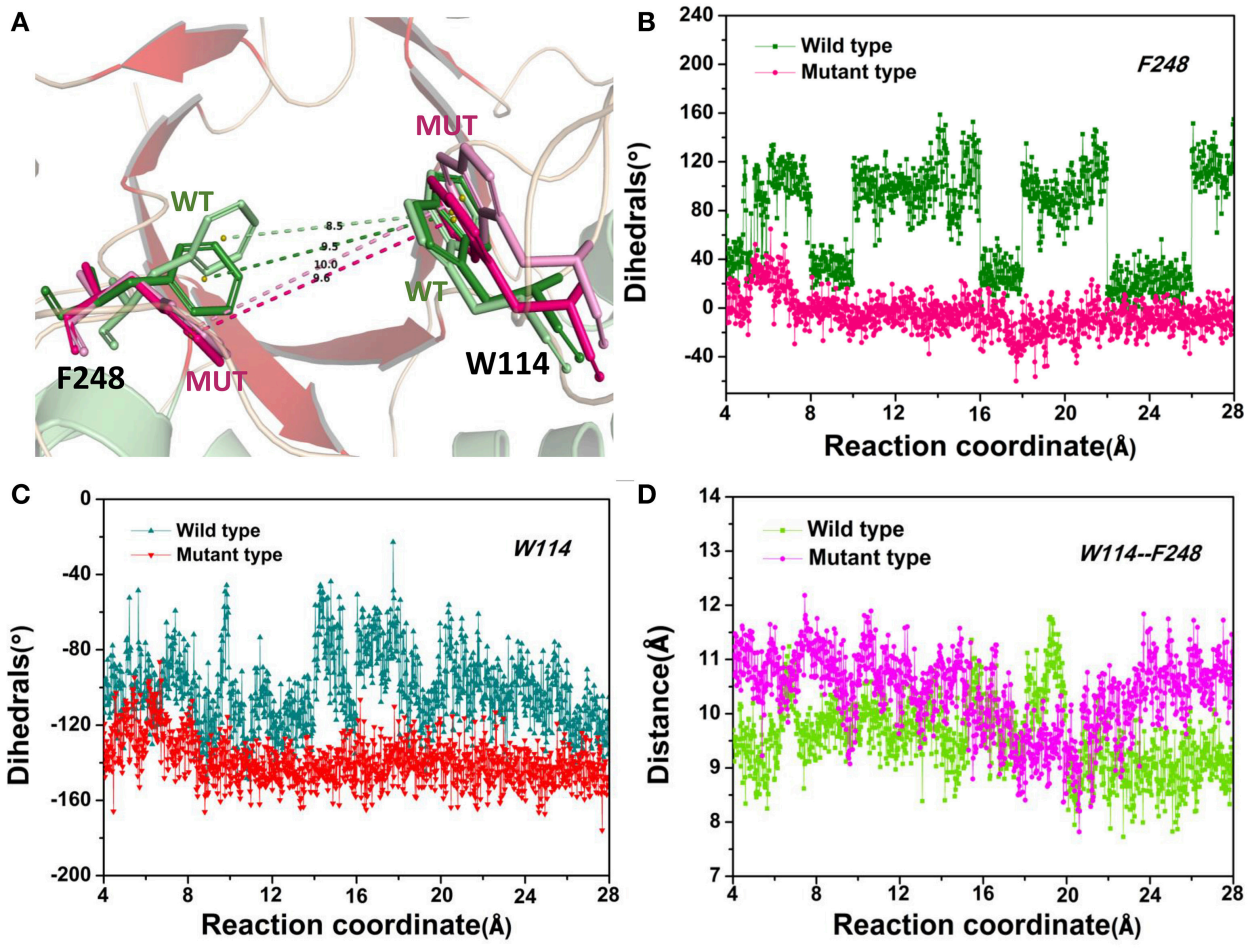
**(A)** The movements of W114 and F248 of the wild-type *Cb*DPEase at 13 Å (light green) and 24 Å (green), of the Y68I/G109P mutant at 13 Å (light pink) and 24 Å (pink). **(B)** The dihedral changes of F248 of the wild-type *Cb*DPEase and Y68I/G109P mutant during ASMD simulations. **(C)** The dihedral changes of W114 of the wild-type *Cb*DPEase and Y68I/G109P mutant during ASMD simulations. **(D)** The distance between the centroid of benzene rings of W114 and F248 in the wild-type *Cb*DPEase and Y68I/G109P mutant.

The D-fructose dissociation from the Y68I/G109P mutant was shown in Figure 10. At 4 Å along the reaction coordinate (Figure 10A), D-fructose was tightly fixed by hydrogen bond interactions with E34, I35, I68, G107, G108, P109, and E246, which were stronger than the wild-type *Cb*DPEase at the identical coordinate. Moreover, D-fructose was also fastened by functional residues in active sites (Y6, E34, I35, G67, G69, G107, G108, E152, and E246) and mutated residues (I68 and P109) through the interatomic contact and combined interactions. Then at 7.5 Å, the D-fructose was wrapped by hydrogen bond interactions with E34, G108, E152, and E246, which were weaker than the wild-type *Cb*DPEase, resulting in the decreased free energy value (Figure 10B). Noticeably, at the same coordinate, the interatomic and combined interactions in the Y68I/G109P mutant were much smaller than that of the wild-type. At 10 Å, the regeneration of interatomic contact, combined with interactions and hydrogen bond interactions in Y6 and I68 (Figure 10C), was characterized by a sudden increase in free energy value. At 13 Å, D-fructose could merely form hydrogen bond interactions with one residue of E246, and the interatomic contact and combined interactions between the substrate and the tunnel residues were also decreased. Thus, the free energy located at a platform level (Figure 10D). After this plateau period of free energy, a rapid increase occured because of the strong hydrogen bond interactions between the substrate and the tunnel residues. For instance, D-fructose could form hydrogen bonds with Y6, E246 and R263 at 15 Å (Figure 10E). After that, at 18 Å, D-fructose formed certain new hydrogen bonds with W114, E158, H188, and F248 (Figure 10F). With the movement of the D-fructose, the substrate formed two donor-π interactions with F248 and W114. As illustrated in Figures 11A–C, the rotation motions of aromatic rings of F248 and W114 along the dissociation in the wild-type *Cb*DPEase and Y68I/G109P mutant were quantified. It was apparent that the benzene ring of the F248 in the wild-type *Cb*DPEase flipped in a great fluctuant way. But in the Y68I/G109P mutant, the dihedral angle of the F248 was stable at around 0°. Similarly, for the dihedrals angle of W114, the angle change of the wild-type was also larger than that of the mutant, and the dihedral angle of the mutant was steady at about 140°. From the structure diagram (Figure 11A), we could clearly see that F248 and W114 located at the entrance tunnel, which could act as the gatekeeper to regulate the opening of tunnel. The planes of benzene rings of F248 and W114 in the Y68I/G109P mutant could be continuously parallel to the stretching direction of the D-fructose, giving rise to the open of the gate of “entrance” in the tunnel. Such open state was favorable for the D-fructose to form the stable donor-π interactions with the benzene rings. Moreover, in the Y68I/G109P mutant, the distance between F248 and W114 in the center of the benzene rings was larger than that of the wild-type, which conformed to the open state of “entrance” in the tunnel (Figures 11A,D). Then, at 25 Å, the free energy value had a rapid increase because of the strong hydrogen bond between D-fructose and residues W114, V116, Y157, E158, and R263 (Figure 10G). Lastly, the free energy was increased continually until the D-fructose was fully dissociated after 28 ns.

We could come to the following conclusions that D-fructose had the stronger binding affinity with the Y68I/G109P mutant than with the wild-type *Cb*DPEase. Moreover, analyses of interactions could enable us to explore the binding/unbinding pathways and provided vital explanations to the catalytic mechanism of *Cb*DPEase at the molecular structure level.

## Conclusions

The catalytic efficiency of the *Cb*DPEase has been enhanced via using the site-directed mutagenesis (Y68I/G109P) technique. To comprehend the structure-activity relationship in the wild-type *Cb*DPEase and Y68I/G109P mutant, we applied molecular modeling methods with protein structure networks for the substrate D-fructose bound to the wild-type *Cb*DPEase and Y68I/G109P mutant, correspondently. Protein structure network analyses and network-based centrality detected a great connectivity between D-fructose and the Y68I/G109P mutant through the high betweenness of several functional residues in the global interaction network. Moreover, during the dissociation of the D-fructose from the Y68I/G109P mutant, the planes of benzene rings of F248 and W114 could be continuously parallel to the stretching direction of the D-fructose. It was equivalent to opening the gate of “entrance” in the tunnel to trigger the formation of the stable donor-π interactions between the D-fructose and the benzene rings around 18 Å. Furthermore, the stronger substrate-protein interactions for the Y68I/G109P mutant were detected in comparison with the wild-type *Cb*DPEase, which was in accordance with the binding free energy and PMF results. We could draw a conclusion that site-directed mutagenesis of Y68 and G109 would improve the substrate-binding affinity of *Cb*DPEase and the opening “entrance” should be responsible for the outstanding catalytic efficiency of the Y68I/G109P mutant.

## Author contributions

JZ wrote and revised this paper. YL and JW prepared the tables. ZY made the Supplementary Materials. YL provided some revision advice. YT and WH provided the ideas and modified the papers.

### Conflict of interest statement

YL, JW, and YT were employed by company COFCO (Jilin) Bio-Chemical Technology Co., Ltd. The remaining authors declare that the research was conducted in the absence of any commercial or financial relationships that could be construed as a potential conflict of interest.
