# Supplementary material for: Adaptive Steered Molecular Dynamics Combined With Protein Structure Networks Revealing the Mechanism of Y68I/G109P Mutations That Enhance the Catalytic Activity of D-psicose 3-Epimerase From Clostridium Bolteae
Source: Front Chem. 2018 Sep 24;6:437. doi: 10.3389/fchem.2018.00437 (PMC6166005; doi:10.3389/fchem.2018.00437)
Supplement: Supplementary file 1 [file Data_Sheet_1.PDF]

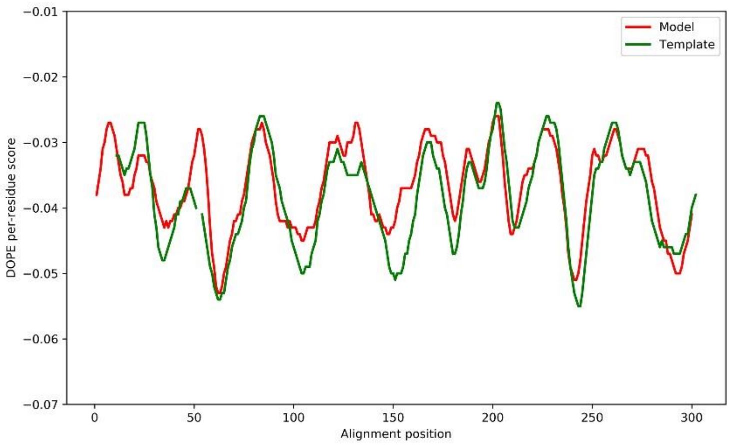

Figure S1. The plotted DOPE score profile for per residue of the modeled structure of *Cb*DPEase (red) and the crystal structure of *Cc*DPEase (green).

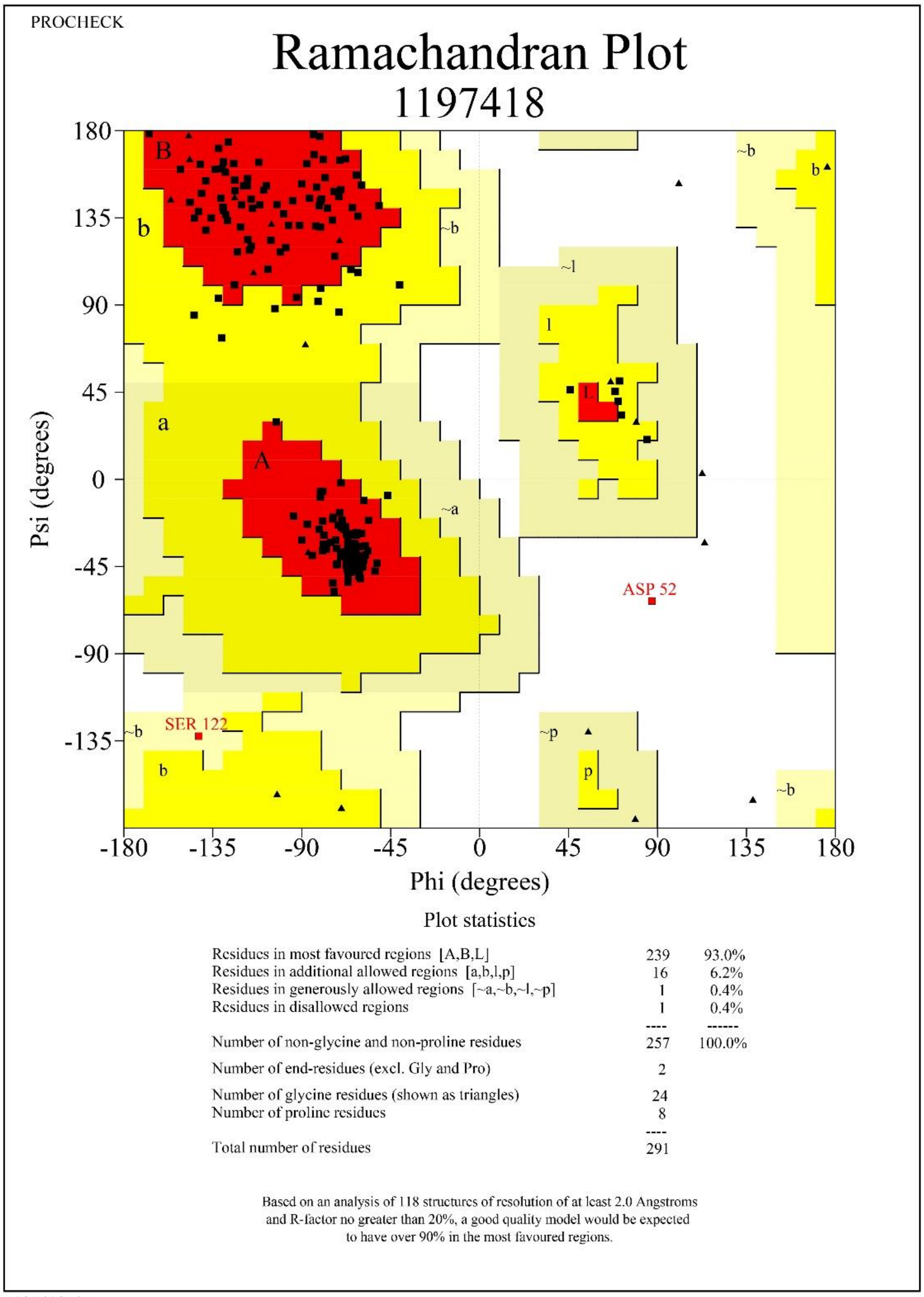

1197418_01.ps

Figure S2. Ramachandran plot of homology modeling structure.

Program: ERRAT2
File: /home/saves/Jobs/8518370/qq_aaaa.pdb_errat.logf

Overall quality factor**: 87.633

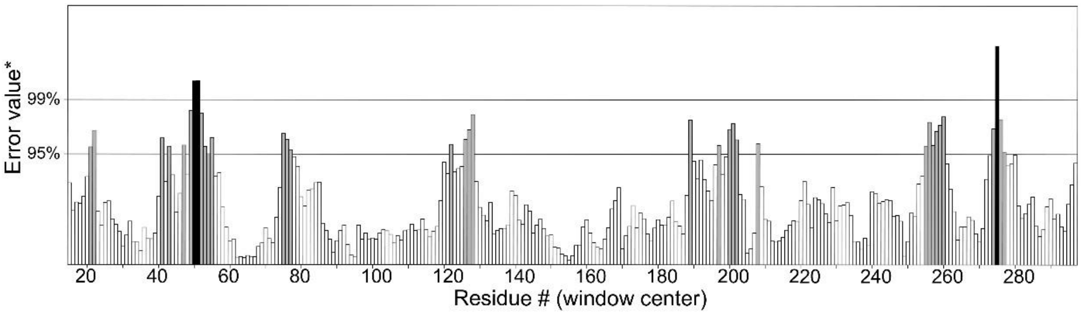

*On the error axis, two lines are drawn to indicate the confidence with which it is possible to reject regions that exceed that error value.

**Expressed as the percentage of the protein for which the calculated error value falls below the 95% rejection limit. Good high resolution structures generally produce values around 95% or higher. For lower resolutions (2.5 to 3A) the average overall quality factor is around 91%.

Figure S3. The average overall quality factor evaluated by ERRAT2 program.

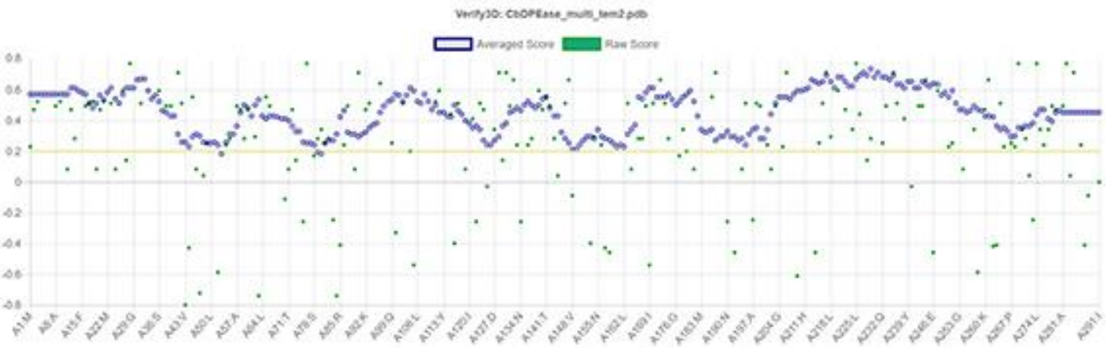

Figure S4. The result of Verify3D validation.

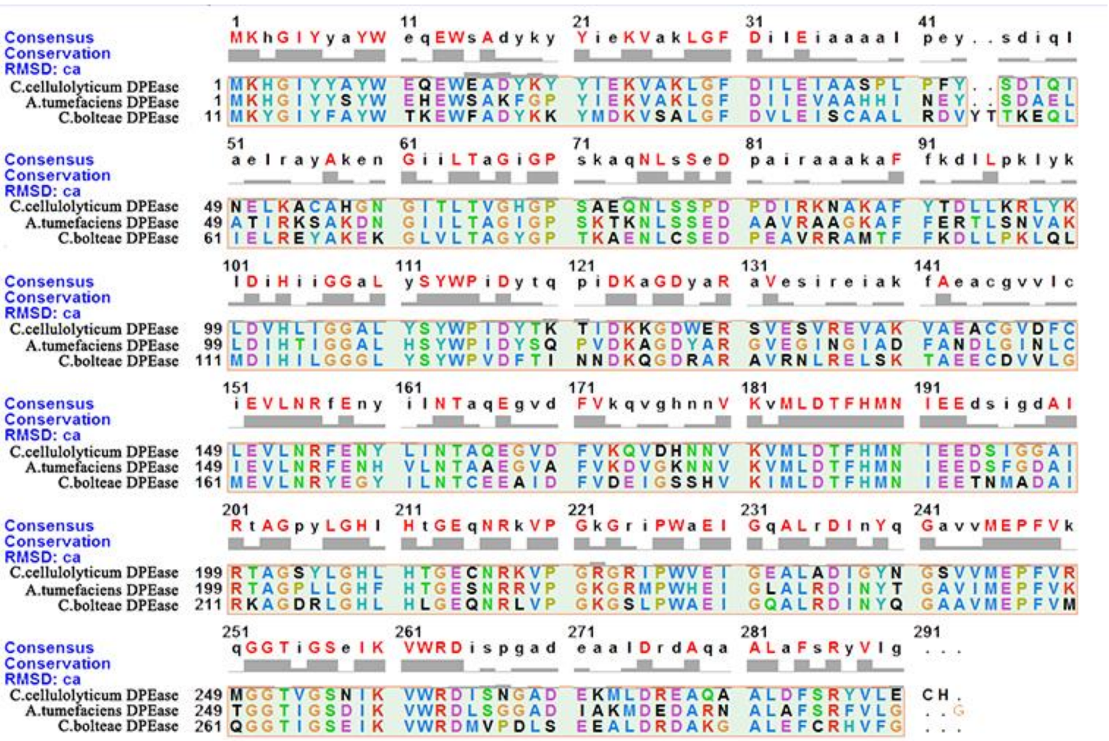

Figure S5. Multiple sequence alignment of DPEases with sequence conservation and RMSD values from different micro-organisms generated with MatchMaker and Match-Align modules are shown with UCSF Chimera. Origins of DPEase enzymes with GenBank accession numbers as follows: *Clostridium cellulolyticum* DPEase, ACL75304; *Agrobacterium fabrum* DPEase ATCC 33970, *Clostridium bolteae* DPEase, ATCC BAA-613.

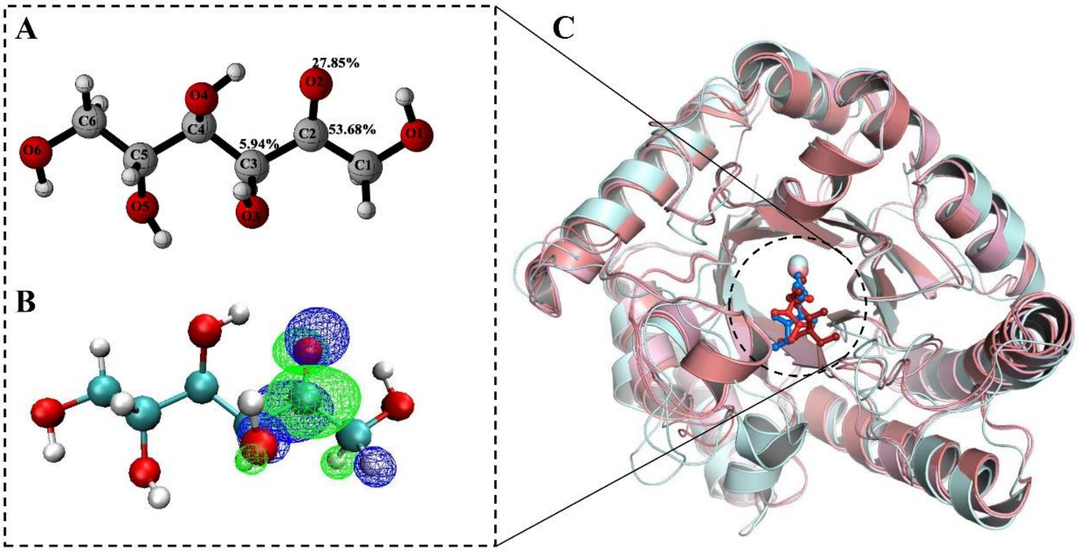

Figure S6. (A), The structure of D-fructose optimized by Gaussian 09 software with B3LYP 6-31+G* set. (B), Natural Bond Orbits (LUMO orbits) of D-fructose. (C), Superimposition of docked ligand conformation (blue) and the reference conformation in the crystal structure (red) were calculated by Autodock Vina.

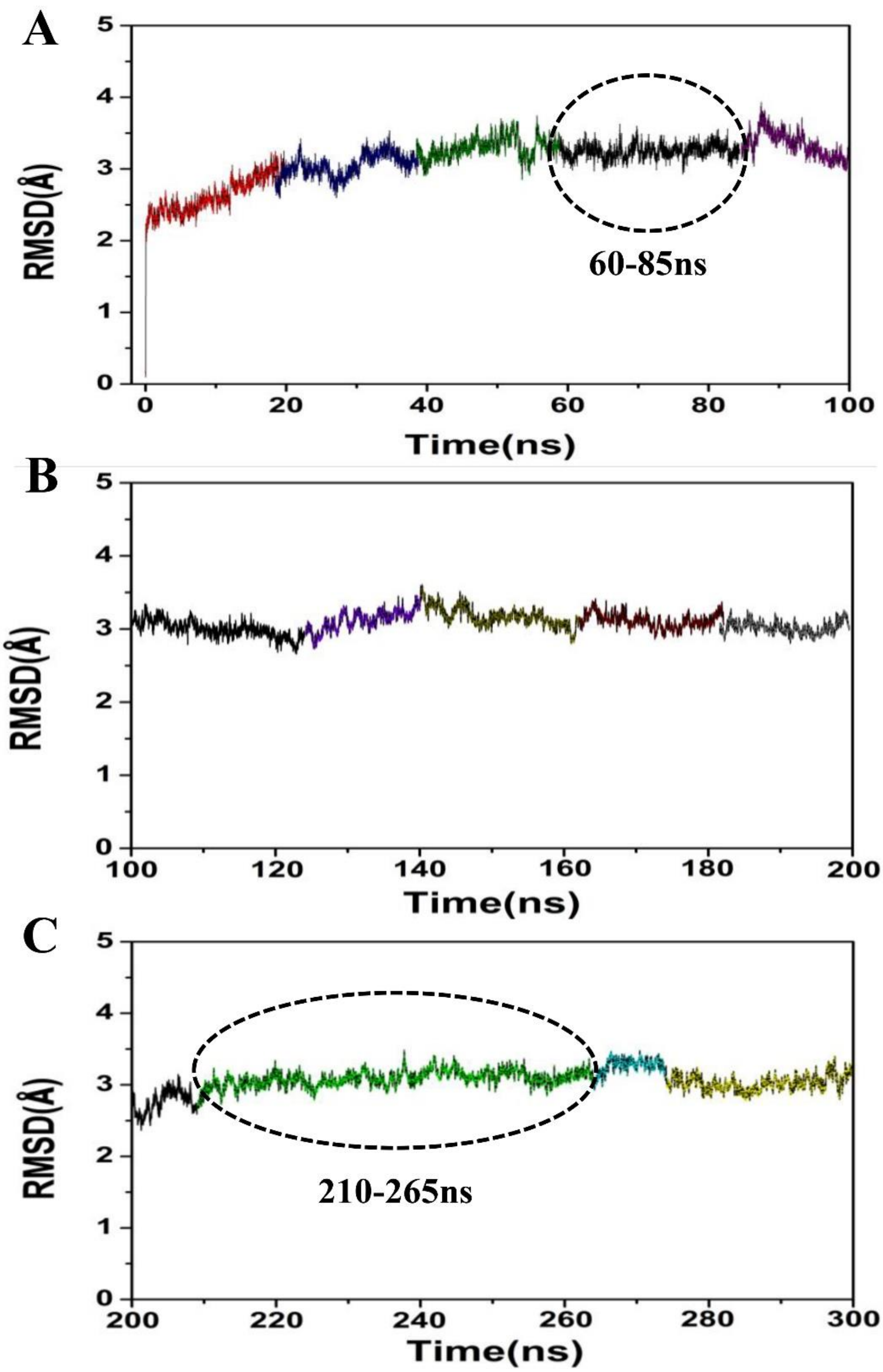

Figure S7. RMSD plot of 300ns MD simulation for model structure.

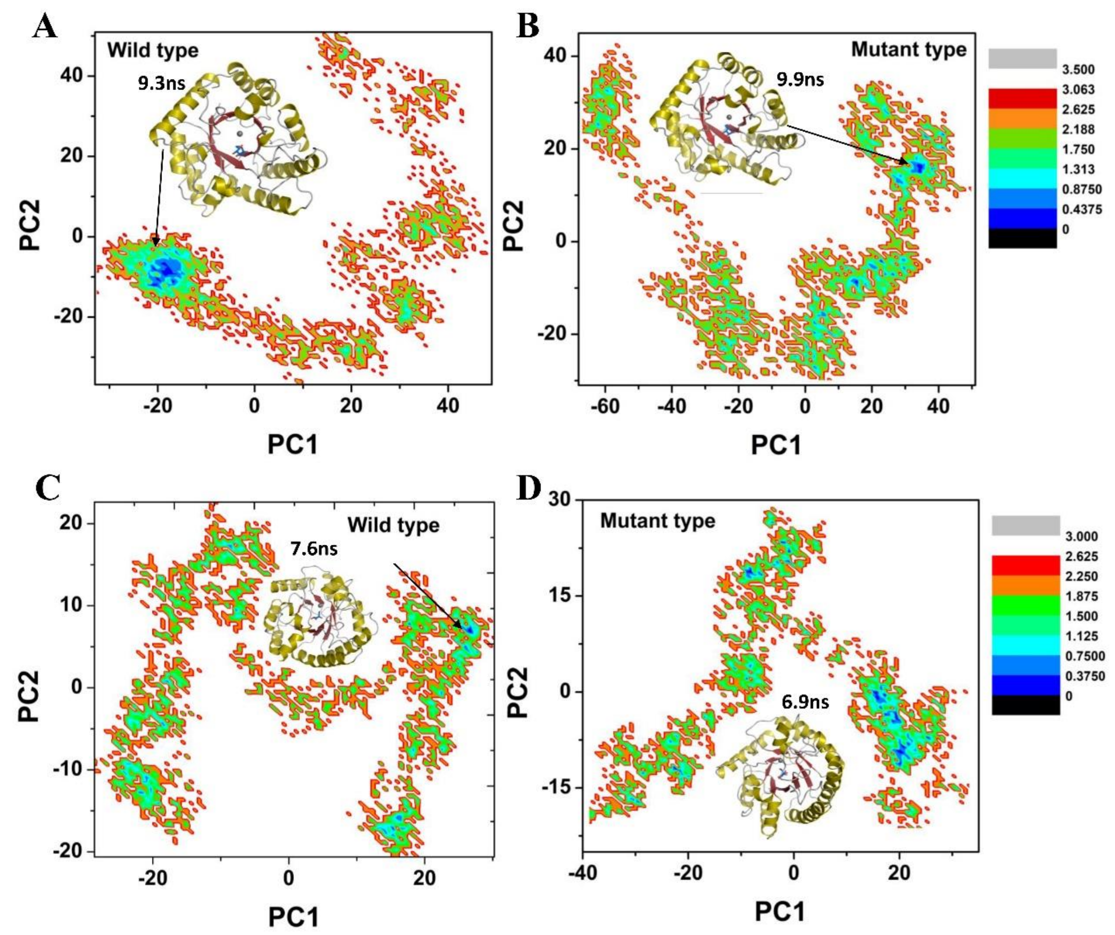

Figure S8. Examples of free energy landscape for the (A), wild-type *Cb*DPEase and (B), Y68I/G109P mutant from the first two equilibrium trajectories; for the (C), wild-type *Cb*DPEase and (D), Y68I/G109P mutant from the latter two equilibrium trajectories.

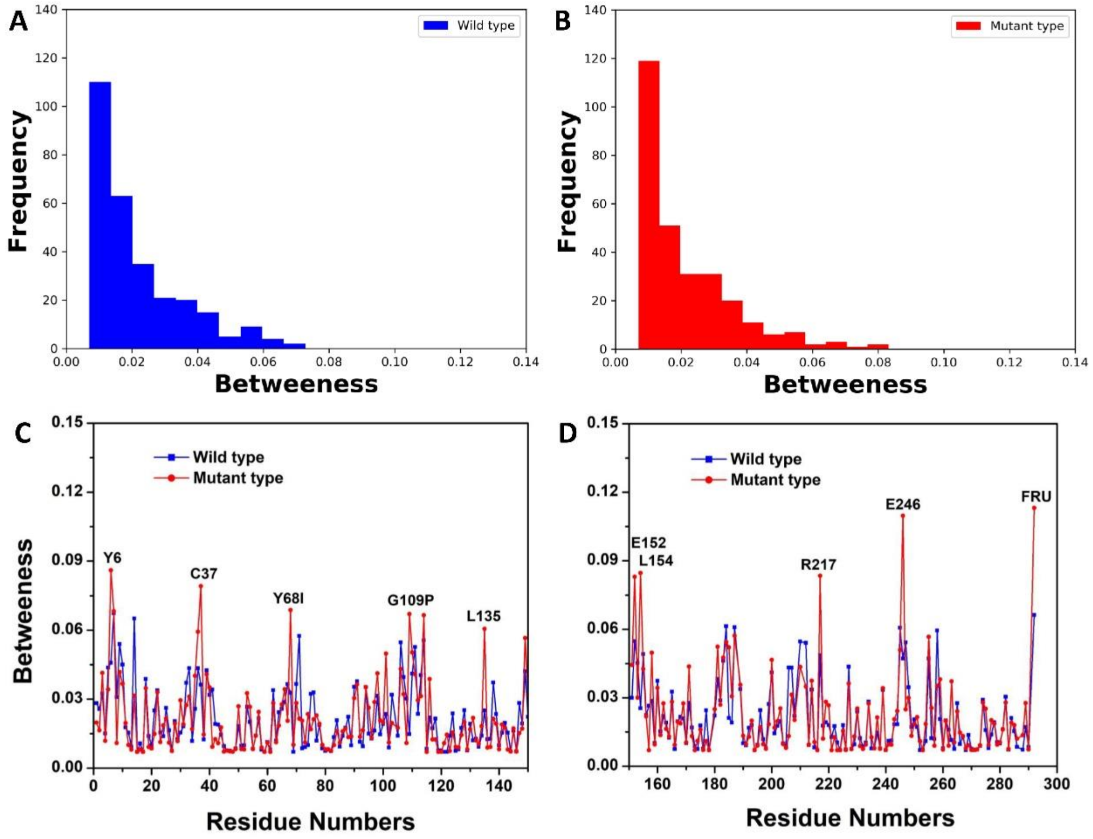

Figure S9. The network analysis of the representative structures from the latter two equilibrium trajectories. The frequency distributions of the betweenness values of the (A), wild-type structure (blue bars), (B), Y68I/G109P mutant type structure (red bars). Residue-based betweenness profiles of the (C), wild-type structure (blue) and (D), Y68I/G109P mutant type structure (red).

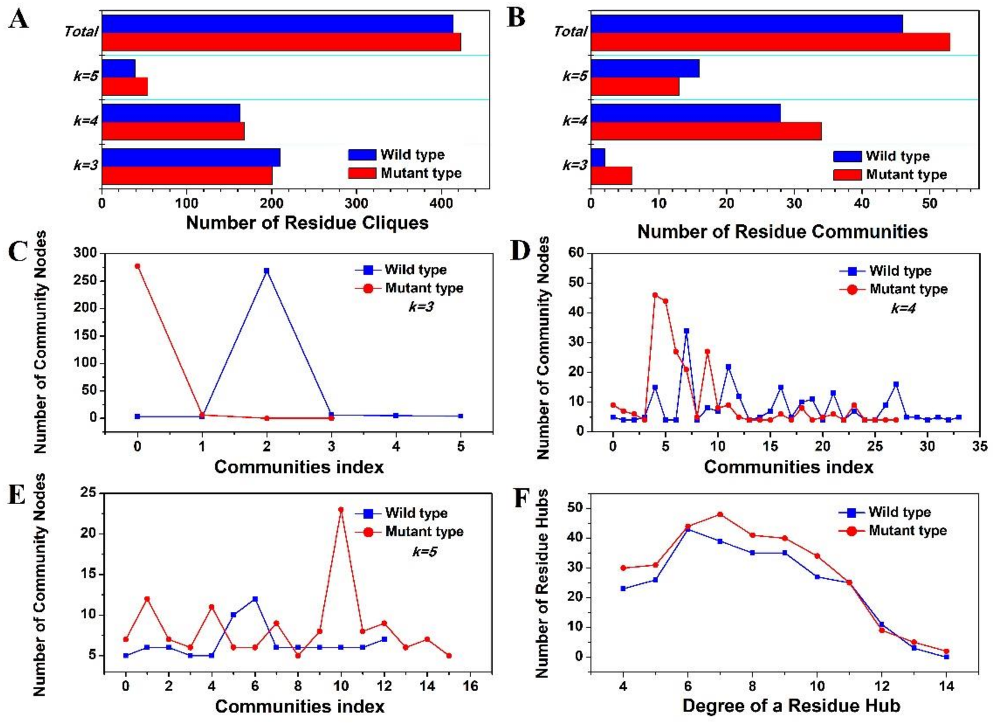

Figure S10. The small-world interaction networks analysis of the representative structures from the latter two equilibrium trajectories. (A), The distribution of cliques in the wild-type structure (blue) and the Y68I/G109P mutant type structure (red). (B), The distribution of communities in the wild-type structure (blue) and the Y68I/G109P mutant type structure (red). The number of community nodes for (C), k=3, (D), k=4, (E), k=5 in the wild-type structure (blue) and the Y68I/G109P mutant type structure (red). (F), The degree distribution of residue hubs in the wild-type structure (blue) and the Y68I/G109P mutant type structure (red).

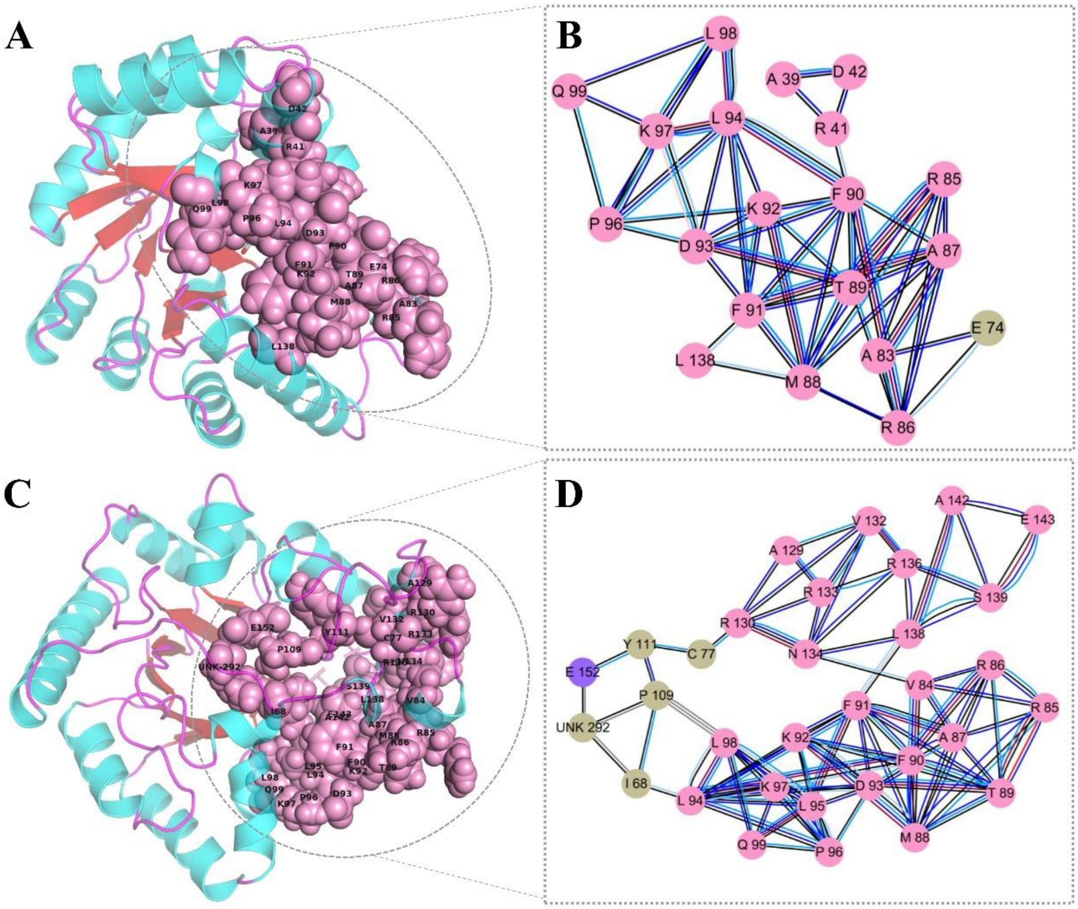

Figure S11. The cluster network analysis of the representative structures from the latter two equilibrium trajectories. (A), General overview and (B), a detailed close-up of the network of cluster in the wild-type structure. (C), General overview and (D), a detailed close-up of the network of cluster in the Y68I/G109P mutant type structure.

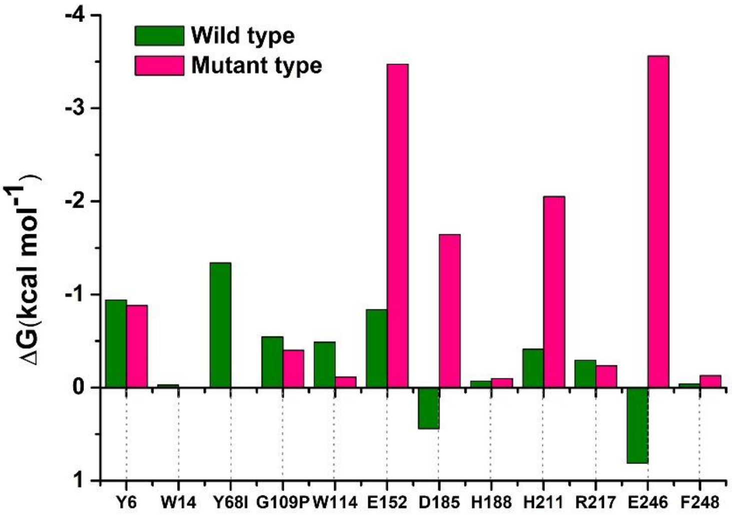

Figure S12. The MM-GB/SA results of the individual residues in the wild-type *Cb*DPEase (green) and Y68I/G109P mutant (pink).

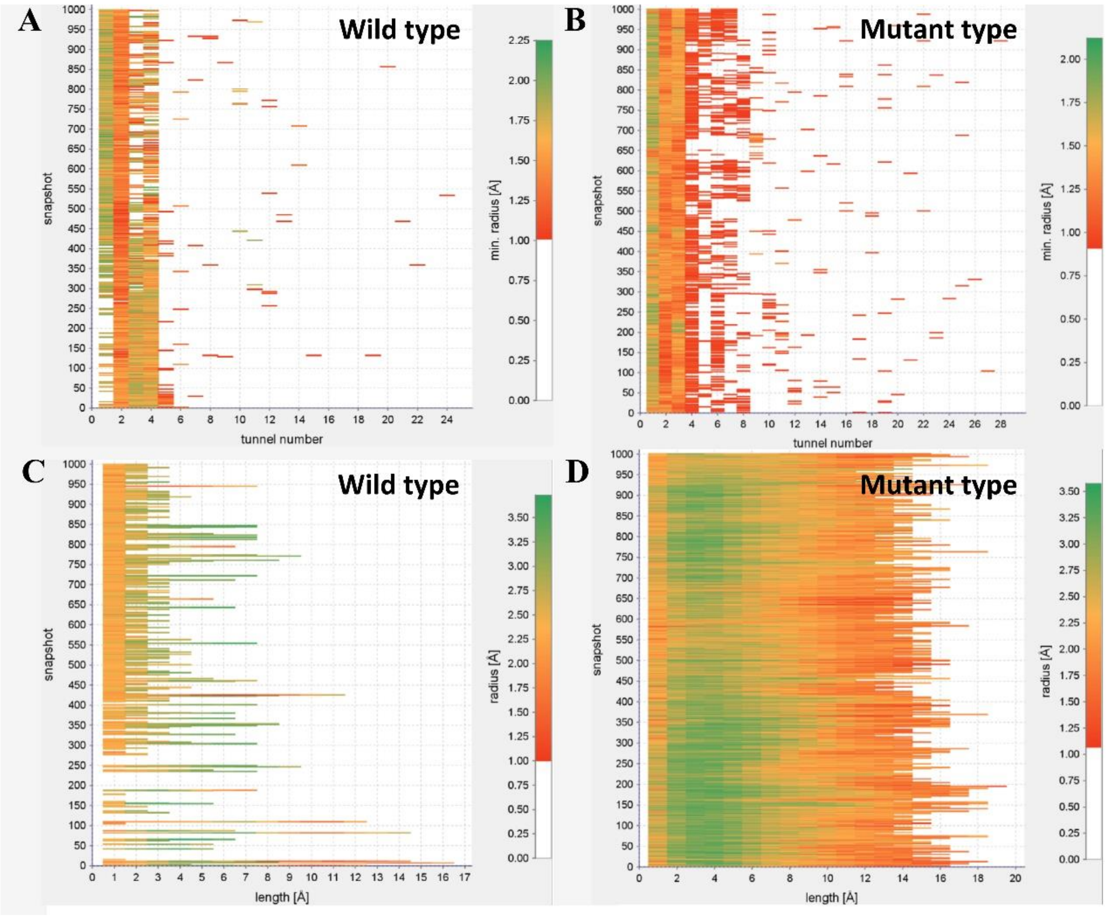

Figure S13. ATH (All Tunnels Heat Map) representation of all tunnels for the (A), wild-type *Cb*DPEase and (B), Y68I/G109P mutant. Each column corresponds to one tunnel, the vertical axis represents time. White spaces denote that a given tunnel is closed at the corresponding timestep. Single Tunnel Heat Maps (STH) representation of the most accessible tunnels (tunnel 1) of the (C), wild-type *Cb*DPEase and (D), Y68I/G109P mutant over time.

Table S1. Energy terms obtained using MM-GB/SA for the D-fructose bound to the wild-type *Cb*DPEase and Y68I/G109P mutant extracted from the latter two equilibrium trajectories (kcal/mol).

System	Wild-type *Cb*DPEase	Y68I/G109P mutant
ΔE_{vdW}	-19.25 ± 3.54	-13.60 ± 3.80
ΔE_{ele}	-56.00 ± 11.17	-75.43 ± 9.50
ΔG_{GB}	63.21 ± 7.95	70.05 ± 5.35
ΔG_{SA}	-3.93 ± 0.17	-3.71 ± 0.14
ΔG_{polar}[a]	7.21	-5.38
$\Delta G_{nonpolar}$[b]	-23.18	-17.31
$\Delta G_{MM\text{-}GB/SA}$[c]	-15.97 ± 3.89	-22.69 ± 4.99
$-T\Delta S$	8.47 ± 3.69	7.83 ± 2.86
ΔG_{bind}[d]	-7.50	-14.86
k_{cat}/K_m (min^{-1} mM^{-1})	59.2 ± 2.9	73.7 ± 6.2

[a] $\Delta G_{polar} = \Delta E_{ele} + \Delta G_{GB}$

[b] $\Delta G_{nonpolar} = \Delta E_{vdW} + \Delta G_{SA}$

[c] $\Delta G_{MM\text{-}GB/SA} = \Delta E_{ele} + \Delta G_{GB} + \Delta E_{vdW} + \Delta G_{SA}$

[d] $\Delta G_{bind} = \Delta G_{MM\text{-}GB/SA} - T\Delta S$

[e] k_{cat}/K_m values obtained from the experimental data (min^{-1} mM^{-1}) (Zhang et al., 2016a).
